# Enhanced Spin-Engineering Photothermoelectric–Enzymatic Catalysis System via Lattice Mismatch-Induced Jahn–Teller Distortion for Tumor Therapy

**DOI:** 10.1007/s40820-026-02175-y

**Published:** 2026-04-09

**Authors:** Pengyu Zang, Meiqi Yang, Chenghao Yu, Rui Zhang, Avez Sharipov, Ruifang Shen, Dan Yang, Shili Gai, Piaoping Yang

**Affiliations:** 1 Key Laboratory of Superlight Materials and Surface Technology, Ministry of Education, College of Materials Science and Chemical Engineering, Harbin Engineering University, Harbin, 150001 People’s Republic of China; 2 Tashkent Pharmaceutical Institute, Ministry of the Health of Uzbekistan, 100015 Tashkent, Republic of Uzbekistan; 3 Laboratory for Space Environment and Physical Sciences, Harbin Institute of Technology, Harbin, 150006 People’s Republic of China

**Keywords:** Spin engineering, Jahn–Teller effect, Multi-enzyme activity, P–n junctions, Photothermoelectric therapy

## Abstract

Oxygen-dependent electrodynamic therapy is hindered by electron–hole recombination and hypoxia. This study provides a heterojunction-induced Jahn–Teller distortion-enhanced spin-engineering Fe_3_O_4_–Ag_2_S nanoplatform to address these limitations. The large interfacial lattice mismatch induces previously unrecognized Jahn–Teller distortions on high-spin Fe sites, modifying d-orbital splitting and enhancing spin-polarized catalytic activity. This lattice–spin–carrier coupling synergistically amplifies catalase-, peroxidase-, and glutathioneox-like pathways. Under near-infrared irradiation, the photothermal effect of Fe_3_O_4_ activates the thermoelectric response of Ag_2_S and drives continuous hot-carrier injection. Thermoelectric fields drive hot holes to boost catalase activity through Jahn–Teller effect-enhanced spin catalysis sites and drive hot electrons to convert O_2 _ to cytotoxic O_2_^.^^−^ and ^1^O_2_ under the Jahn–Teller distortion, promoting and forming a self-amplifying catalytic loop. Fine structure characterization and density functional theory calculations collectively verify strain-driven Fe–O bond differentiation and spin-state reconfiguration. The heterojunction achieves potent thermoelectric–enzyme co-catalysis with 95% tumor inhibition under near-infrared irradiation and supports dual-mode imaging. This work establishes a framework for designing high-performance photothermal–thermoelectric catalysts through crystal field/spin-state modulation in p–n heterojunctions, synergistically boosting multi-enzyme activity and catalytic efficiency for hypoxia-resistant therapy

**Supplementary Information:**

The online version contains supplementary material available at 10.1007/s40820-026-02175-y.

## Highlights

- A Fe_3_O_4_–Ag_2_S p–n heterojunction is engineered to induce interfacial Jahn–Teller distortion, which modifies d-orbital splitting and enhances spin-polarized catalytic activity for synergistic tumor therapy.
- The photothermal-driven thermoelectric effect generates an electric field that facilitates directional charge separation, concurrently boosting multiple enzyme-mimicking activities for enhanced catalytic therapy.
- Density functional theory calculations confirm that the Jahn–Teller distortion optimizes the d-orbital and spin state of Fe^3+^, providing a theoretical basis for the superior thermoelectric and enzymatic performance.

**Supplementary Information:**

The online version contains supplementary material available at 10.1007/s40820-026-02175-y.

## Graphical abstract

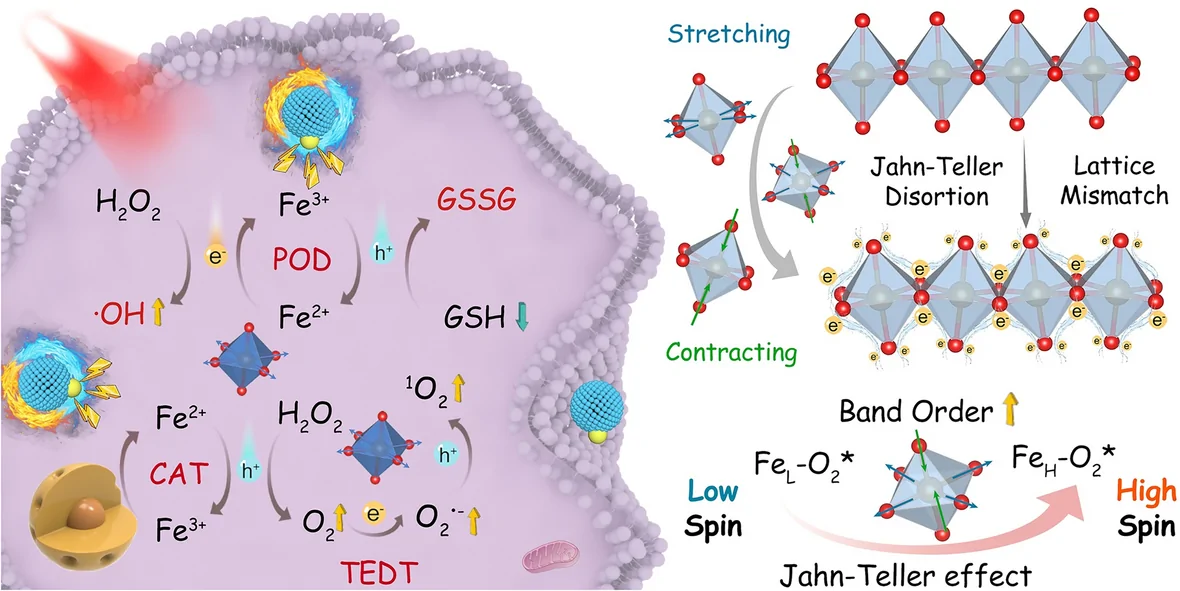

## Introduction

The rapid advancement of nanomedicine has enabled nanodynamic therapies to overcome the limitations of conventional therapy, such as imprecision and systemic toxicity [1–3]. Classical nanodynamic therapies include chemodynamic therapy [4, 5], photodynamic therapy [6, 7], and others [8–13]. Thermoelectric dynamic therapy (TEDT) [14, 15], a promising approach, exhibits advantages such as deep tissue penetration and efficient reactive oxygen species (ROS) generation [16, 17]. TEDT relies on thermoelectric semiconductor materials [18, 19] and possesses two critical challenges: inefficient carrier separation due to rapid electron–hole recombination and hypoxia in the tumor microenvironment [20]. To address carrier recombination, strategies focus on improving thermoelectric material properties by reducing thermal conductivity and enhancing power factors [21, 22]. However, their inherent trade-off complicates optimization. Heterojunction engineering has emerged as a promising strategy, as interfacial potentials mitigate carrier recombination [23, 24]. Specifically, p–n heterojunctions generate robust internal electric fields at the interface due to significant charge redistribution, enabling unidirectional carrier transport [25–27]. The second challenge for TEDT is the hypoxic tumor microenvironment [28]. Common strategies to alleviate hypoxia involve integrating electrocatalytic oxygen evolution reaction (OER) components or catalase (CAT)-mimetic enzymes [29, 30]. However, as a four-electron transfer process, OER suffers from sluggish kinetics [29]. While the two-electron pathway inherent to CAT-like active materials offers distinct advantages in tumor treatment, their catalytic efficiency under physiological conditions remains a critical challenge.

Electronic structure modulation is one of the most effective approaches for enhancing CAT-like performance [31, 32]. The spin states and magnetic properties of electrocatalysts influence energy levels associated with radical interactions [33]. Spin transitions between singlet H_2_O_2_ and triplet O_2_ require a minimum energy barrier of ≈1 eV [34]. Thus, strategies promoting spin-state transitions could significantly accelerate CAT-like kinetics [35]. According to Hund's rule, the number and degeneracy of electronic orbitals strongly correlate with the electron spin polarization. Transition metal oxides, commonly employed in the nanozymes [36], often demonstrate split three-dimensional orbitals into two degenerate sets to minimize the energy: The *t*_2*g*_ orbitals (*d*_xy_, *d*_yz_, and *d*_xz_) and the *e*_*g*_ orbitals (*d*_*x*_^*2*^_*-y*_^*2*^ and *d*_*z*_^*2*^). This splitting induces inherent instability in some octahedral structures [37]. Consequently, these transition metal compounds typically undergo spontaneous geometric distortions to minimize total system energy, a phenomenon recognized as the Jahn–Teller distortion [38]. Recent studies have revealed that the Jahn–Teller effect can effectively modulate catalytic activity [39, 40]. Pronounced Jahn–Teller effects eliminate the degeneracy of *e*_*g*_ orbitals, inducing significant distortions along the *z*-axis or *xy*-plane, accompanied by lattice destabilization [41]. This process enhances crystalline disorder and compressive strain while stabilizing antibonding orbitals [42], thereby promoting catalytic activity.

Recent advances in heterojunction engineering for photothermal/catalytic therapy have primarily focused on optimizing interfacial charge separation and band alignment to enhance photothermal conversion and ROS generation, effectively addressing issues such as carrier recombination and hypoxia through improved electronic dynamics [43–45]. In contrast to these conventional electronic band engineering strategies [20, 24], the present work introduces a distinct design paradigm. Herein, we constructed a Fe_3_O_4_–Ag_2_S p–n heterojunction to leverage a large interfacial lattice mismatch, which is designed to induce pronounced Jahn–Teller distortion. This structural distortion serves as the primary driver to reconfigure the local crystal field and modulate the spin state of catalytic metal centers, establishing a lattice–spin–activity cascade. This approach provides an additional, structurally derived dimension for catalytic regulation, moving beyond conventional charge carrier dynamics to enable direct coupling between interfacial strain, spin polarization, and enzymatic activity for enhanced TEDT and multi-enzyme catalytic therapy.

**Fig. 1:**
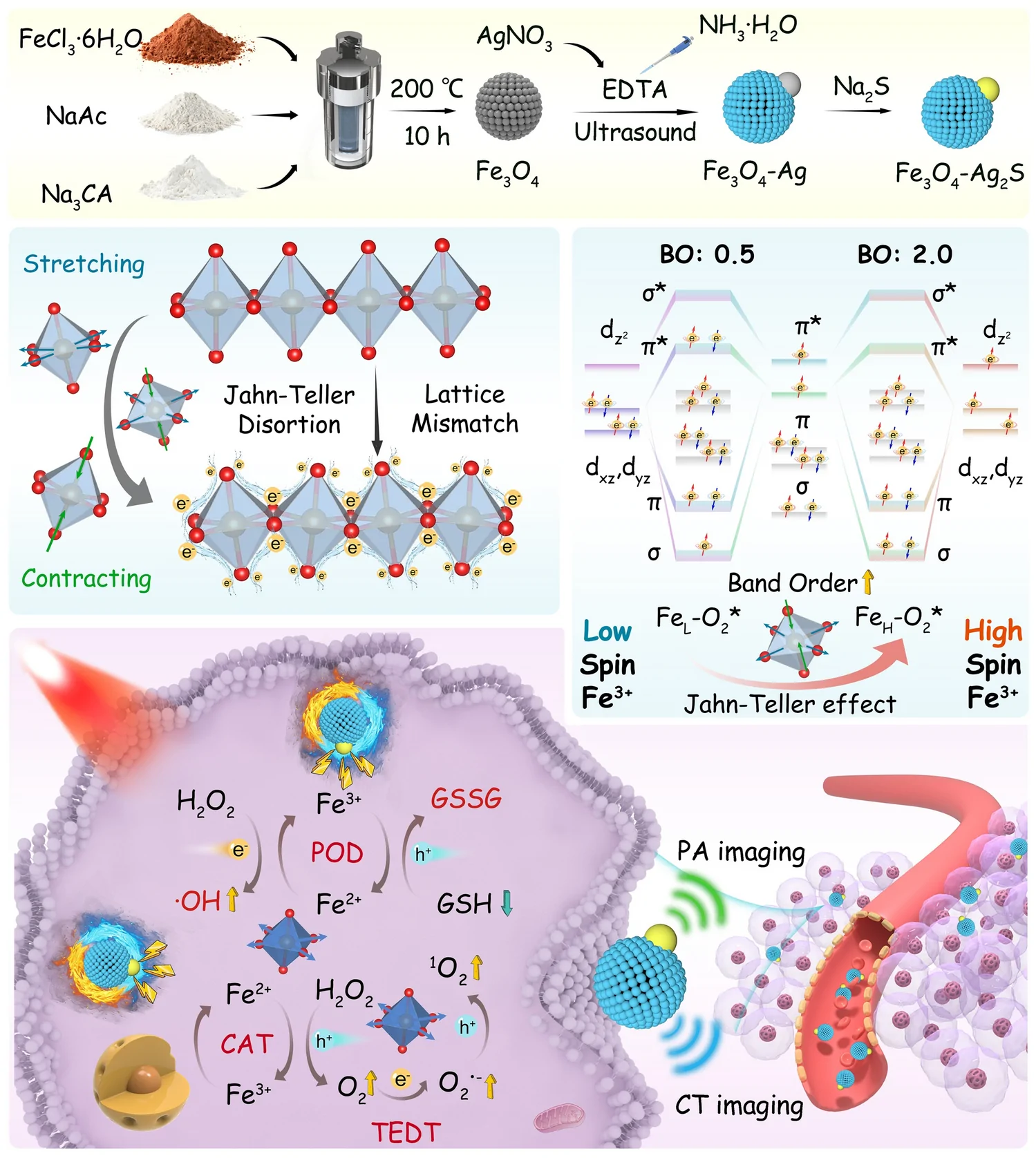
Schematic illustration of Fe_3_O_4_–Ag_2_S and its antitumor mechanism of Jahn–Teller effect-enhanced spin-engineered TEDT–enzyme catalytic therapy combination therapy

Specifically, as illustrated in Fig. 1, Fe_3_O_4_ nanoparticles are hydrothermally synthesized, followed by Ag nanoparticle growth and sulfidation to form Fe_3_O_4_–Ag_2_S. The thermoelectric response of Ag_2_S, activate by 808 nm laser, drives unidirectional conduction of the p–n junction, enhancing electron–hole separation. Under thermoelectric fields, hot holes injected from Ag_2_S to Fe_3_O_4_ promote CAT-like activity, enhanced by Jahn–Teller effect. The molecular orbital theory further confirms this enhancement. While hot electrons utilize CAT-generated O_2_ to produce O_2_^.^^−^, establishing a positive feedback loop between enzyme activity and TEDT. Moreover, X-ray absorption near-edge structure (XANES) analysis and density functional theory (DFT) calculations validate these findings and clarify the origin of Jahn–Teller distortions. It also confirms the spin-enhanced ^1^O_2_ generation. Furthermore, hot holes oxidize glutathione (GSH), while Fe_3_O_4_-derived hot electrons reduce H_2_O_2_ to •OH, boosting peroxidase (POD)-like activity. In vitro and in vivo experiments demonstrate the high synergistic therapeutic efficacy of Fe_3_O_4_–Ag_2_S. Additionally, the incorporation of Ag_2_S enabled computed tomography (CT) imaging, while the photothermal performance of Fe_3_O_4_–Ag_2_S facilities photoacoustic (PA) imaging. In summary, the Fe_3_O_4_–Ag_2_S system efficiently integrates TEDT with multi-enzyme catalytic therapy through heterojunction engineering-induced crystal field and spin-state modulation.

## Experimental Section

### Synthesis of Fe_3_O_4_–Ag_2_S

####  Synthesis of Fe_3_O_4_ NPs

First, 3.25 g FeCl_3_·6H_2_O, 1.3 g sodium citrate, and 6.0 g sodium acetate were added to 100 mL glycol and stirred. The mixture is then transferred and sealed into a Teflon-lined stainless steel autoclave (capacity 200 mL). After the reaction at 200 °C for 10 h, the black product was collected by the external magnetic field and washed with deionized water and ethanol 3 times, respectively. Finally, the obtained Fe_3_O_4_ NPs were vacuum-dried for 12 h.

####  Ag NPs Decoration

Subsequently, Fe_3_O_4_ was functionalized with ethylenediaminetetraacetic acid disodium salt (2Na-EDTA). Briefly, 100 mg of as-prepared Fe_3_O_4_ was dispersed in 20 mL of deionized water, followed by the addition of 100 mg of 2Na-EDTA. The suspension was subjected to ultrasonic treatment for 15 min to ensure sufficient surface coordination. The obtained Fe_3_O_4_@EDTA was separated by centrifugation and dried under vacuum at 50 °C. For the deposition of Ag nanoparticles, a silver–ammonia complex solution was prepared by dissolving 30 mg of AgNO_3_ in 30 mL of water and adding approximately 5 mL of 5% ammonia until complete complexation. Subsequently, 50 mg of Fe_3_O_4_@EDTA was introduced into the silver–ammonia solution and ultrasonicated at room temperature for 30 min. The resulting Ag-decorated Fe_3_O_4_ was magnetically separated, thoroughly rinsed with ultrapure water and ethanol, and finally dried in a vacuum oven at 60 °C.

####  Sulfurization

20 mmol of Na_2_S·9H_2_O was dissolved in 10 mL of deionized water, which was recorded as solution A. The Ag–Fe_3_O_4_ product was dispersed in 30 mL of deionized water, recorded as solution B. Solution A was slowly added drop by drop into solution B under vigorous stirring. Subsequently, the reaction product was collected with magnets, washed several times with ultrapure water and ethanol, and then dispersed in deionized water.

### DFT Calculations

All DFT calculations were performed using the Vienna Ab initio Simulation Package (VASP). All energetics of metal oxides were calculated using the DFT with the Hubbard U framework (DFT + U) to account for strongly localized d electrons for Fe and Ag. A plane-wave cutoff energy of 520 eV was adopted, and Brillouin zone sampling was performed using a 2 × 2 × 1 Monkhorst–Pack *k*-point grid. All atoms were fully relaxed with the energy convergence tolerance of 10^–5^ eV per atom, and the final force on each atom was < 0.05 eV Å^−1^.

The adsorption energy was calculated using the following formula:

1 $$\Delta G_{ads} = \Delta E_{ads} + E_{ZPE} - T\Delta S$$

where ads = (*H_2_O_2_, *OH, O*, O_2_*), $\Delta {E}_{\mathrm{ZPE}}$ is the zero-point energy change, and ΔS is the entropy change. In this work, the values of $\Delta {E}_{\mathrm{ZPE}}$ and ΔS were obtained by vibration frequency calculation.

### Calculation of Photothermal Conversion Efficiency of Fe_3_O_4_–Ag_2_S Nanoparticles

The heating and cooling temperatures of the Fe_3_O_4_–Ag_2_S (500 μg mL^–1^) were recorded. The photothermal conversion efficiency (*η*_*T*_) was calculated using the following formula:

2 $$\eta _{T} {\kern 1pt} = {\kern 1pt} \frac{{{\mathrm{hS}}\left( {T_{{{\mathrm{max}}}} - T_{{{\mathrm{surr}}}} } \right) - Q_{{{\mathrm{dis}}}} }}{{{\mathrm{I}}\left( {1 - 10^{{ - {\mathrm{A}}_{\lambda } }} } \right)}}$$

where *h* is the thermal conversion efficiency of the system, *S* is the surface area of the container, *T*_max_ is the equilibrium temperature of the sample solution, *T*_surr_ represents the surrounding ambient temperature, *I* is the power density of the 808-nm continuous laser (0.4 W cm^−2^), and *A*_*λ*_ is the absorbance of Fe_3_O_4_–Ag_2_S solution at *λ* = 808 nm. The absorbance of 500 μg mL^–1^ Fe_3_O_4_–Ag_2_S at 808 nm is 1.7. Additionally, *Q*_dis_ represents the heat loss due to the light absorption of the container itself, and it was determined as *Q*_dis_ = (5.4 × 10^−4^) *I*. To calculate *hS*, another equation was introduced:

3 $$hS{\kern 1pt} = {\kern 1pt} \frac{{{\mathrm{mC}}_{{{\mathrm{water}}}} }}{{\tau _{s} }}$$

where m is the sample’s mass, *C*_water_ is water’s heat capacity (4.2 J g^−1^ K^−1^), and *τ*_*s*_ is the sample system time constant, which is calculated by the equation *t* = − *τ*_*s*_ ln(*θ*). t is the instantaneous time when the temperature drops and *θ* is the driving force temperature, which can be calculated by the following formula: *θ* = (*T* − *T*_surr_) / (*T*_max_ − *T*_surr_), where *T* is the instantaneous temperature, *T*_surr_ is the ambient temperature (30 °C), and *T*_max_ is the highest steady-state temperature (70.7 °C). By substituting these values into these equations, the 808 nm *η* of Fe_3_O_4_–Ag_2_S was calculated as 46.33%.

### Evaluation of O_2_^.^^−^ Generation

Fe_3_O_4_–Ag_2_S (500 μg mL^–1^) and dihydrorhodamine 123 (DHR123) (20 μM) were dispersed in 3.0 mL of phosphate buffer solution (PBS) (pH = 6.5). After different laser irradiation durations (0.4 W cm^–2^ for 0, 1, 3, and 5 min) or water bath cycle (80/30 °C and 60/30 °C for 0, 1, 3, and 5 min, each temperature is maintained for 30s), the luminescence of R123 at 525 nm under 412 nm excitation at given time points was collected by PL spectrofluorometer. Under the same conditions, we used dimethylpyridine nitroxide (DMPO) as the trapping agent of O_2_^.^^−^, and the generation of O_2_^.^^−^ was proved by the electron spin resonance (ESR) spectrum.

### Evaluation of •OH Generation

Fe_3_O_4_–Ag_2_S (500 μg mL^–1^) and 3,3',5,5'-tetramethylbenzidine (TMB) (3 mg mL^–1^) were dispersed in 3.0 mL of PBS (pH = 6.5). Then H_2_O_2_ was added. (Working concentration was 1 mM.) After different laser irradiation durations (0.4 W cm^–2^ for 1, 2, 3, 4, and 5 min) or water bath cycle (60/30 °C for 0, 1, 3, and 5 min, each temperature is maintained for 30s), the absorbance was recorded by a UV-1601 spectrophotometer to quantify the generation rate of •OH Under the same conditions, we used DMPO as the trapping agent of •OH, and the generation of •OH was proved by the ESR spectrum. We further detected the production of •OH with TA. Fe_3_O_4_–Ag_2_S solution (500 μg mL^–1^) containing terephthalic acid (TA) (5 mM) and NaOH (2 mM) was irradiated with 808-nm laser irradiation (0.4 W cm^–2^ for 1, 2, 3, and 5 min), and record fluorescence spectrum (*λ*_ex_ = 315 nm). The changes of fluorescence intensity (*λ*_ex_ = 315 nm, *λ*_em_ = 445 nm) at given time point.

### POD-like Enzyme Kinetics

To evaluate the POD-like kinetics, Fe_3_O_4_, Fe_3_O_4_–Ag, Fe_3_O_4_–Ag_2_S (500 μg mL^–1^), and TMB (10 mM) were added to 3 mL of PBS solution after laser irradiation for 5 min, and H_2_O_2_ was added at different concentrations (final concentrations of 15.125, 31.25, 61.5, 125, and 250 mM). Then, the absorbance at 652 nm after different reaction times was measured, and the Michaelis–Menten constant was determined based on the Michaelis–Menten equation (Eq. 4). The maximum reaction velocity (*V*_max_) and the Michaelis–Menten constant (*K*_*m*_) were determined from the corresponding Lineweaver–Burk plots (Eq. 5). The [*S*] in Eqs. 1 and 2 is the concentration of the substrate.

4 $$\frac{{V_{{{\mathrm{max}}}} \left[ S \right]}}{{K_{m} + \left[ S \right]}}$$

5 $$\frac{1}{{V_{0} }} = \frac{{K_{m} }}{{V_{{{\mathrm{max}}}} }} \cdot \frac{1}{\left[ S \right]} + \frac{1}{{V_{{{\mathrm{max}}}} }}$$

The catalytic kinetics of Fe_3_O_4_–Ag_2_S at 60 °C were determined with a similar procedure.

### GSH Depletion Evaluation

Fe_3_O_4_–Ag_2_S (500 μg mL^–1^) were mixed with GSH (5 mM), H_2_O_2_ (0.1 mM), and 5,5'-Dithiobis-(2-nitrobenzoic acid) (DTNB) with a final concentration of 0.3 × 10^–3^ M to detect –SH of GSH in PBS solution after different treatments (808-nm irradiation, 60/30 °C water bath cycle). The absorbance spectrum was recorded via a UV–Vis spectrophotometer.

### Electrochemical Characterizations

The photo-/thermal current and Mott–Schottky curve assay of Fe_3_O_4_, Ag_2_S, and Fe_3_O_4_–Ag_2_S were measured by an electrochemical analyzer (CHI650E) in the Na_2_SO_4_ aqueous solution (0.1 M) using Pt wire as the counter electrode, and Ag/AgCl as a reference electrode, respectively. To prepare the working electrode, 500 µg of the samples was coated on the glassy carbon electrode using Nafion/ethanol solution (volume-to-volume ratio *V*_Nafion_ / *V*_ethanol_ = 1:9). The *E*_fb_ for the normal hydrogen electrode (NHE) can be calculated as follows:

6 $$\begin{array}{*{20}c} {E_{fb} \left( {vs NHE} \right) = E_{fb} \left( {vsAg/AgCl} \right) + E_{Ag/AgCl} + 0.0592 \times pH} \\ \end{array}$$

where *E*_Ag*/*AgCl_ is 0.199 V at 25 °C and the pH of the Na_2_SO_4_ electrolyte is 7.

### Band Gap Detection

The band gap of Fe_3_O_4_, Ag_2_S, and Fe_3_O_4_–Ag_2_S was calculated according to the Kubelka–Munk equation based on the UV–Vis absorption spectra as follows:

7 $$\alpha {\mathrm{h}}\nu = A\left( {h\nu - E_{g} } \right)^{2}$$

where *α*, h, *ν*, and *A* are the absorption coefficient, Planck’s constant, frequency of the incident light, and a constant, respectively.

#### In Vitro Cellular Uptake

To examine cellular uptake, confocal laser scanning microscopy (CLSM) was employed. Fluorescein isothiocyanate (FITC)-labeled Fe_3_O_4_–Ag_2_S nanoparticles were prepared by vigorously stirring an ethanol solution (5 mL) containing FITC (2 mg mL⁻^1^) and Fe_3_O_4_–Ag_2_S (200 μg mL⁻^1^) for 6h. 4T1 cells were seeded in 6-well plates and cultured overnight to allow adhesion. The cells were then incubated with 1 mL of FITC-labeled Fe_3_O_4_–Ag_2_S for varying durations (0.5, 1, 3, and 5h), followed by washing several times with PBS. Subsequently, the cells were stained with Hoechst 33,342 for 15 min and, according to the manufacturer’s instructions, counterstained with Lyso-Tracker (100 nM). After fixation with 2.5% glutaraldehyde (1 mL) and further PBS washes, fluorescence imaging was performed using a Leica TCS SP8 system.

#### Detection of Intracellular ROS Production

2',7'-Dichlorodihydrofluorescein diacetate (DCFH-DA) was used to detect the intracellular ROS generation ability of the Fe_3_O_4_–Ag_2_S. 4T1 cells were seeded onto 6-well plates overnight and then were treated with (1) control, (2) 808 nm (0.4 W cm^−2^), (3) Fe_3_O_4_, (4) Fe_3_O_4_ + NIR (808 nm, 0.4 W cm^−2^), (3) Fe_3_O_4_–Ag_2_S, and (4) Fe_3_O_4_–Ag_2_S + NIR (808 nm, 0.4 W cm^−2^). The concentration of Fe_3_O_4_ and Fe_3_O_4_–Ag_2_S was 200 μg mL^−1^. Subsequently, DCFH-DA was used as a ROS fluorescence probe by incubating for 15 min. Then, the cells were washed with PBS several times and stained with Hoechst 33,342 for 15 min. Afterward, 1 mL of glutaraldehyde (2.5%) was added to fix the morphology of cells and then washed with PBS. Finally, the intracellular fluorescence was monitored using a Leica TCS SP8 instrument.

#### Mitochondrial Integrity Assay

We used the 5,5′,6,6′-tetrachloro-1,1′,3,3′-tetraethyl-imidacarbocyanine iodide (JC-1) to detect intracellular mitochondrial membrane potential. The cells were then treated with different groups: (1) control, (2) 808 nm (0.4 W cm^−2^), (3) Fe_3_O_4_, (4) Fe_3_O_4_ + NIR (808 nm, 0.4 W cm^−2^), (3) Fe_3_O_4_–Ag_2_S, and (4) Fe_3_O_4_–Ag_2_S + NIR (808 nm, 0.4 W cm^−2^). Then Hoechst 33,342 was stained for 15 min. Afterward, glutaraldehyde (2.5%) was added to fix the morphology of cells and then washed with PBS. Finally, the cells were imaged using a Leica TCS SP8 instrument.

#### Live/Dead Staining

4T1 cells were treated with different groups: (1) control, (2) 808 nm (0.4 W cm^−2^), (3) Fe_3_O_4_, (4) Fe_3_O_4_ + NIR (808 nm, 0.4 W cm^−2^), (3) Fe_3_O_4_–Ag_2_S, and (4) Fe_3_O_4_–Ag_2_S + NIR (808 nm, 0.4 W cm^−2^). The concentration of Fe_3_O_4_ and Fe_3_O_4_–Ag_2_S was 200 μg mL^−1^. After incubating for 5h, the treated cells were stained with calcein AM (2 μM) and propidium iodide (PI) (4 μM) for 15 min.

#### Apoptosis Detection Assay

For a quantitative analysis of apoptosis-mediated cell death, 4T1 cells were seeded in a 6-well plate (1 × 10^5^ cells per well) and cultured overnight. After treated with different groups: (1) control, (2) 808 nm (0.4 W cm^−2^), (3) Fe_3_O_4_, (4) Fe_3_O_4_ + NIR (808 nm, 0.4 W cm^−2^), (5) Fe_3_O_4_–Ag_2_S, and (6) Fe_3_O_4_–Ag_2_S + NIR (808 nm, 0.4 W cm^−2^), the concentration of Fe_3_O_4_ and Fe_3_O_4_–Ag_2_S was 200 μg mL^−1^. After incubating for 5 h, the cells were trypsinized, washed, and quantified based on apoptosis via an annexin V-FITC/PI apoptosis detection kit using a flow cytometer.

#### In Vivo Pharmacokinetic Evaluation

Female Balb/c mice were subcutaneously transplanted with 4T1 cancer cells (100 μL), and the tumors grew to 80 mm^3^ before the experiment was started. All mice were then intravenously injected with Fe_3_O_4_–Ag_2_S (10 mg kg^−1^), and at 1, 3, 6, 12, and 24 h post-injection, the mice were sacrificed. Major organs (hearts, livers, spleens, lungs, and kidneys) and tumors were dissected, rinsed with PBS, weighed, and dissolved with HNO_3_ and H_2_O_2_ mixed solution. The biodistributions in different organs and tumors were calculated as the Ag percentage of the injected dose per gram of tissue by emission spectrometer (ICP–OES). In addition, female Balb/c mice (*n* = 3) were tail intravenous (*i.v.*) injection with Fe_3_O_4_–Ag_2_S. For blood circulation, Balb/c mice (*n* = 3) were *i.v.* injection with Fe_3_O_4_–Ag_2_S (10 mg kg^−1^). At given time points (0, 0.0833, 0.1667, 0.25, 0.5, 1, 2, 4, 8, 10, 12, and 24 h), 100 μL of blood samples was collected and the amount of Ag was quantitated by ICP–OES.

#### In Vitro and In Vivo PA Imaging Performance

The in vitro and in vivo PA imaging performances were measured using a Vevo LAZR-X system. Briefly, Fe_3_O_4_–Ag_2_S was dissolved in PBS solutions with various concentrations (31.25, 62.5, 125, 500, and 1000 μg mL^−1^) and then recorded by a Vevo LAZR-X system. The wavelength in the scanning process was set from 680 to 970 nm. For the in vivo PA imaging analysis, the 4T1 tumor-bearing mice were imaged before injection to acquire the background signal of the mice. Afterward, the mice were *i.v.* injection with Fe_3_O_4_–Ag_2_S (10 mg kg^−1^). At 0, 1, 3, 6, 12, and 24 h post-injection, the mice were anesthetized for PA imaging at an exciting wavelength of 800 nm.

#### In vitro and In Vivo CT Imaging

In vitro CT imaging was performed by dispersing Fe_3_O_4_–Ag_2_S at different concentrations (0.625, 1.25, 2.5, 5, 10, and 20 mg mL^−1^) in PBS. For in vivo CT imaging, the tumor-bearing mice were *i.v.* injected with Fe_3_O_4_–Ag_2_S solution (10 mg kg^−1^). At 0, 1, 3, 6, 12, and 24 h post-injection, CT scanning was performed using a small-animal X-ray CT imaging system (Quantum GX, PerkinElmer).

#### In vivo Synergetic Photothermal-Induced Nanocatalytic Tumor Therapy of Fe_3_O_4_–Ag_2_S

To establish the tumor model, the female Balb/c mice (17 ± 1 g, 5 weeks) were established by subcutaneous inoculation of 4T1 cancer cells (2 × 10^6^, 100 μL) at the right posterior of the mice. Further experiments began when the tumor volume had reached approximately 80 mm^3^. Moreover, as a mature and classic method, the subcutaneous transplantation model has significant advantages in terms of reproducibility. In that case, we calculated the effect size (Cohens’ d) use the following formula:

8 $$d{ } = \frac{{\left( {X1{ } - { }X2} \right)}}{{\sigma_{{{\mathrm{pooled}}}} }}$$

where the *d* was represented Cohens’ d value, the *X*_*1*_ and *X*_*2*_ were represented the average of group 1 and 2, and the *σ*_pooled_ was represented the combined sample standard deviation of group 1 and 2. The Cohens’ d value of control group and Fe_3_O_4_–Ag_2_S + NIR group was to be approximately 1.04, which is considered a ‘large effect’ (typically defined as *d* > 0.8). Although the sample size is small, the observed large effect value has to some extent increased the credibility of our results. The mice were randomly divided into 6 groups (*n* = 5 per group): (1) control, (2) 808 nm (0.4 W cm^−2^), (3) Fe_3_O_4_, (4) Fe_3_O_4_ + NIR (808 nm, 0.4 W cm^−2^), (5) Fe_3_O_4_–Ag_2_S, and (6) Fe_3_O_4_–Ag_2_S + NIR (808 nm, 0.4 W cm^−2^). The mice in each group were then given *i.v.* injection with Fe_3_O_4_ or Fe_3_O_4_–Ag_2_S (10 mg kg^−1^, 0.2 mL), and saline for group (1) and (2). Afterward, their body weights and relative tumor volumes were recorded every day to evaluate the performance of the treatment.

#### Tumor Volume and Inhibition Rate Calculation

After *i.v.* injection, each mouse was weighed and tumor volume was measured daily, and the formula for calculating tumor volume is: Tumor volume (V) = length × width × height /2. Tumor inhibition rate = [1 – (RTV_Exp_ / RTV_Control_)] × 100%, where RTV_Exp_ is the relative tumor volume of the experimental group, and RTV_Control_ is the relative tumor volume of the control group. RTV = *V*_*t*_ / *V*_0_. *V*_*t*_: tumor volume at the end of an experimental cycle. *V*_0_: tumor volume at the beginning of the experiment. In addition, we provided the standard deviation of each treatment group to demonstrate the effectiveness of the treatment. The standard deviation of (1) control, (2) 808 nm (0.4 W cm^−2^), (3) Fe_3_O_4_, (4) Fe_3_O_4_ + NIR (808 nm, 0.4 W cm^−2^), (5) Fe_3_O_4_–Ag_2_S, and (6) Fe_3_O_4_–Ag_2_S + NIR (808 nm, 0.4 W cm^−2^) were 1.1462, 1.46154, and 1.78602.

#### Statistical Analysis

Unpaired two-tailed Student’s *t*-test was used to compare the statistical significance between the two data groups. Quantitative data were indicated as mean ± S. D. Asterisks were used to represent significant differences (n. s.: no significance, *p < 0.05, **p < 0.01, and ***p < 0.001). The statistical analysis was performed by using IBM SPSS Statistics 25 software. Sample sizes (*n*) are labeled in the figure captions of all statistical plots.

## Results and Discussion

### Synthesis and Structural Characterization

**Fig. 2:**
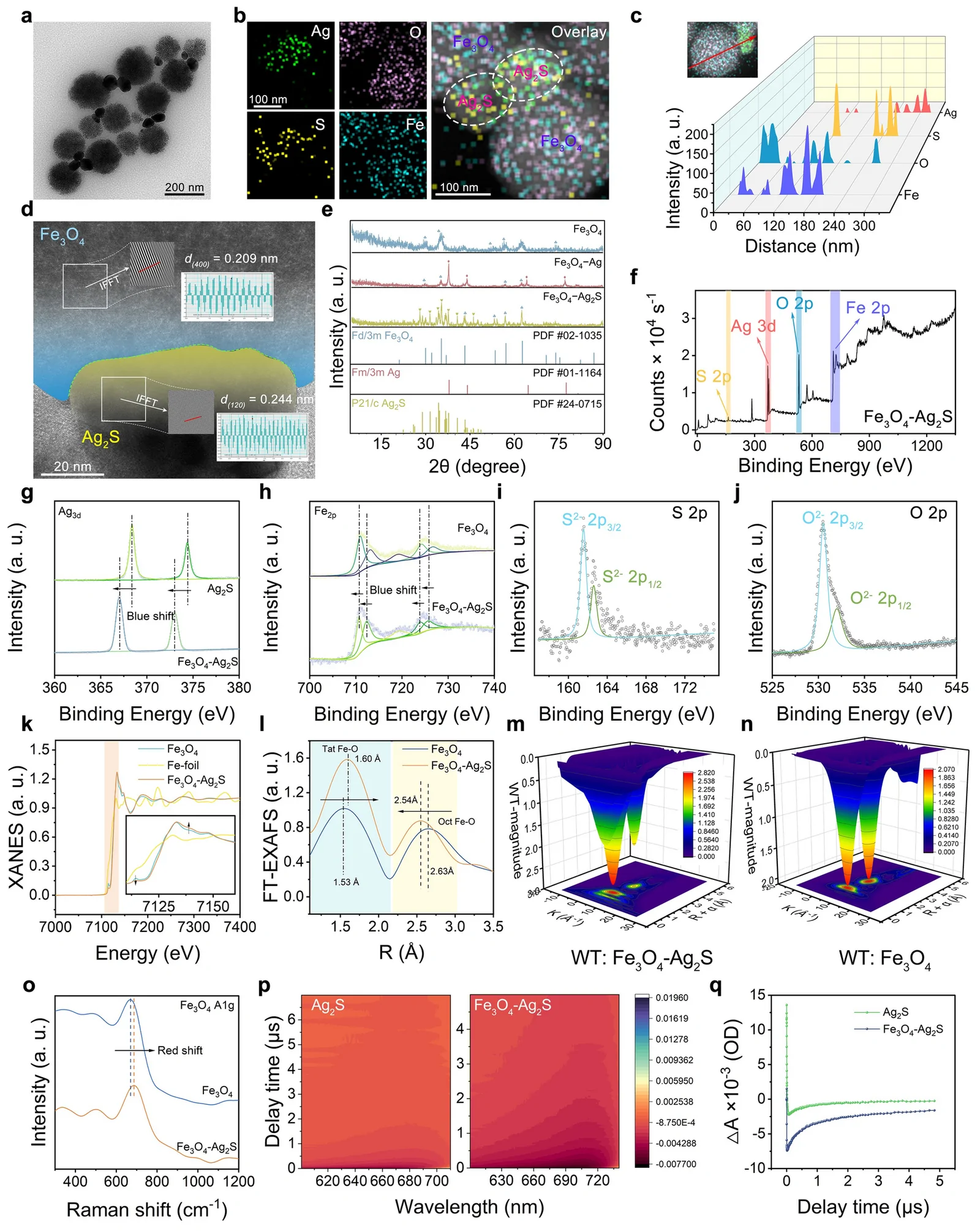
Compositional and structural characterization. **a** TEM image of Fe_3_O_4_–Ag_2_S. **b** HAADF, elemental distribution mapping, and **c** line scan element distribution. **d** HRTEM images and inverse Fourier transform analysis. **e** XRD patterns of Fe_3_O_4_, Fe_3_O_4_–Ag, and Fe_3_O_4_–Ag_2_S. **f** XPS spectra of Fe_3_O_4_–Ag_2_S. High-resolution XPS spectra of **g** Ag 3*d*, h Fe 2*p*, **i** S 2*p*, and **j** O 2*p*. **k** XANES spectra of the Fe foil, Fe_3_O_4_, and Fe_3_O_4_–Ag_2_S NPs. **l** FT-EXAFS spectra of the Fe foil, Fe_3_O_4_, and Fe_3_O_4_–Ag_2_S NPs. WT-EXAFS plots for **m** Fe_3_O_4_–Ag_2_S and **n** Fe_3_O_4_ NPs. **o** Raman and **p** transient absorption spectra of Ag_2_S and Fe_3_O_4_–Ag_2_S. **q** Absorbance variation of Ag_2_S and Fe_3_O_4_–Ag_2_S at 700 nm

To synthesize Fe_3_O_4_–Ag_2_S heterojunctions, Fe_3_O_4_ nanoparticles were first prepared using a one-step hydrothermal method. As illustrated in Fig. S1, the Fe_3_O_4_ nanoparticles exhibited a uniform size of approximately 200 nm in diameter. Subsequently, Ag nanoparticles were deposited on the surfaces via the in situ reduction of AgNO_3_ on the surfaces of the Fe_3_O_4_ nanoparticles. Figure S2a, b demonstrates that a single Ag nanoparticle was successfully formed on each Fe_3_O_4_ nanoparticle. The Fe_3_O_4_–Ag_2_S junction was then obtained through sulfidation with Na_2_S. The morphology of the final product, depicted in Figs. 2a and S2c, d, indicated that the sulfidation process did not alter the heterojunction structure. High-angle annular dark-field (HAADF) imaging and elemental mapping, as shown in Fig. 2b, clearly distinguish the Fe_3_O_4_ and Ag_2_S regions. Furthermore, the transmission electron microscopy (TEM) images after incubation in the culture medium for 48 h are shown in Fig. S2e. The images reveal that the structure remained intact.

To further characterize the structure, a randomly selected particle was analyzed for its elemental linear distribution, as shown in Fig. 2c. The line scan profiles confirmed the heterogeneous structure consisting of Fe_3_O_4_ and Ag_2_S. High-resolution transmission electron microscopy (HRTEM) further verified the formation of Fe_3_O_4_–Ag_2_S heterojunction, revealing distinct lattice fringes with interplanar spacings of 0.209 and 0.244 nm, corresponding to the (400) plane of Fe_3_O_4_ and (120) plane of Ag_2_S (Fig. 2d). The X-ray diffraction (XRD) pattern presented in Fig. 2e further confirmed that the final product consisted of Fe_3_O_4_ (PDF#02–1035) and Ag_2_S (PDF#24–0715). To further characterize the surface chemical states of Fe_3_O_4_–Ag_2_S for subsequent evaluation of its catalytic properties, X-ray photoelectron spectroscopy (XPS) analyses were conducted, with the survey spectrum shown in Fig. 2f. Characteristic spectral lines corresponded to S, Ag, O, and Fe were identified. The high-resolution XPS spectra for each element were further analyzed, as illustrated in Fig. 2g–j. Notably, the Fe 2*p* spectrum depicted in Fig. 2h revealed coexistence of Fe^2+^ and Fe^3+^, essential for the subsequent Fenton catalysis. In addition, shifts in Fe 2*p* binding energies and changes in satellite peak features indicated modifications in the local electronic environment and ligand field strength of Fe centers. Such ligand field modulation and Fe–O bond distortion may influence in spin polarization and high-spin/low-spin energetics. Furthermore, the binding energy shifts observed for Ag 3*d* and Fe 2*p* suggested changes in the chemical environment, providing further evidence for epitaxial growth rather than simple electrostatic adsorption.

To further investigate the internal fine structure of the heterojunction, X-ray absorption near-edge structure (XANES) and extended X-ray absorption fine structure (EXAFS) measurements were taken. As shown in Fig. 2k, compared with standard Fe_3_O_4_, the oxidation state of Fe in the heterojunction exhibited only a slight increase, with no abrupt change, which can be clearly attributed to the formation of the heterostructure. Quantitative fitting parameters containing coordination numbers, bond distances, and Debye–Waller factors derived from the EXAFS analysis are provided in Table S1, which further substantiated the observed structural distortion. However, the coordination environment of Fe differed from that of the standard, as reflected by variations in the absorption features upward shift before 7125 eV and downward shift from 7125 to 7150 eV. Notably, this change in coordination number might result from the large lattice mismatch during heterostructure formation. The linear mismatch was calculated using the following formula:

9 $$f = \frac{{\left| {a_{1} - a_{2} } \right|}}{{a_{2} }} \times 100\%$$

where *f* is the mismatch degree, a_1_ and a_2_ are the lattice parameters of Ag_2_S and Fe_3_O_4_ in the interfacial plane (001). The lattice mismatch degrees along a and b directions were 49.7% and 17.6%, respectively. Such a significant mismatch could account for the observed change in coordination number. As shown in Fig. S2f, g, the change in interplanar spacing caused by this large mismatch was captured by HRTEM. This phenomenon also indicated that Ag_2_S grown epitaxially on Fe_3_O_4_, rather than through simple electrostatic adsorption. Furthermore, to investigate the effect of lattice mismatch on the crystal structure, the EXAFS spectra were subjected to Fourier transform (FT) analysis. As shown in Fig. 2l, the two peaks in the FT-EXAFS corresponded to the Fe–O bonds at octahedral and tetrahedral sites, respectively.

The FT-EXAFS results revealed that the average Fe–O bond length at the octahedral sites decreased, while that at the tetrahedral sites increased, which suggesting the possible occurrence of a Jahn–Teller distortion. The wavelet transforms (WT) analyses shown in Fig. 2m visualized more intuitively the variations in Fe–O bonding at different coordination sites. Furthermore, Raman spectroscopy was employed to examine the changes in Fe–O bond length. Based on group theory and lattice vibration analysis of the spinel structure, the normal-mode motion description of the FeO_4_ tetrahedron involves symmetric stretching of the oxygen atoms along the A_1_g of the Fe–O bond [46, 47]. As shown in Fig. 2o, the A1g vibrational mode of Fe_3_O_4_–Ag_2_S exhibited a redshift compared with that of Fe_3_O_4_, further indicated an overall elongation of the tetrahedral site Fe–O bond length, and supporting the occurrence of a Jahn–Teller distortion. Given the well-known suppression of electron–hole recombination in heterojunctions and the electron–hole separation effect induced by Jahn–Teller activity, transient absorption spectroscopy was conducted for Fe_3_O_4_–Ag_2_S and Ag_2_S, as shown in Fig. 2p. Further analysis of the photobleaching region around 700 nm (Fig. 2q) revealed a slower recovery for Fe_3_O_4_–Ag_2_S, confirming its highly efficient electron–hole separation capability. In addition, the aqueous stability was analyzed using zeta potential. As shown in Fig. S3, the high surface potential confirmed the good aqueous dispersion stability of the nanoparticles. To enable the in vivo application of Fe_3_O_4_–Ag_2_S, its colloidal stability was evaluated in culture media containing 10% serum at pH 6 and 7 to mimic physiologically relevant conditions. As shown in Fig. S4, Fe_3_O_4_–Ag_2_S maintained a consistent hydrodynamic diameter of approximately 150 nm with a polymer dispersity index (PDI) below 0.3 throughout a 7-day monitoring period, indicating good resistance to aggregation and serum-induced destabilization. This stability formed an essential prerequisite for reliable in vivo performance and underpinned the subsequent pharmacokinetic, biodistribution, and therapeutic studies.

### TEDT and Enzyme Catalytic Characterization

Photothermal performance is essential for the activation of TEDT. Here, the photothermal performance of Fe_3_O_4_–Ag_2_S was evaluated in a slightly acidic phosphate-buffered saline (PBS) solution, as shown in Fig. S5. Fe_3_O_4_–Ag_2_S exhibited efficient photothermal conversion under 808-nm laser irradiation (Fig. S5a). At a 1 mg mL^−1^ concentration, the temperature reached ~ 61 °C with a maximum heating rate of 1.5 °C s^−1^ after 200 s, establishing a sufficient temperature gradient for TEDT activation. Repeated near-infrared (NIR) cycles confirmed stable photothermal performance (Δ*T* ≈ 40 °C, *η* = 46.33%, Fig. S5b–d). It is worth noting that we studied the photothermal response of Fe_3_O_4_. As shown in Fig. S5e, its photothermal behavior was similar to that of Fe_3_O_4_–Ag_2_S, prompting us to further investigate the differences in their enzymatic and thermoelectric catalytic activities under the same conditions. In addition, the Seebeck coefficient, electrical conductivity, and thermal conductivity of Fe_3_O_4_–Ag_2_S were measured, and the corresponding figure of merit (*zT*) values were calculated at different temperatures. As shown in Fig. S6, the Fe_3_O_4_–Ag_2_S heterojunction exhibited a moderate electrical conductivity and relatively low thermal conductivity. This was a desirable property for thermoelectric materials, as it helped maintain a significant temperature gradient across the material, which was the driving force for the thermoelectric effect. Interfacial scattering between Fe_3_O_4_ and Ag_2_S phases likely contributes to this suppressed thermal transport. The observed catalytic enhancement arises from two distinct effects: (i) thermoelectric effects, where internal fields generated by carrier separation under a temperature gradient activate catalysis [48]; and (ii) thermal enhancement, where reaction kinetics accelerate solely due to photothermal heating. The following analyses aim to disentangle these contributions.

**Fig. 3:**
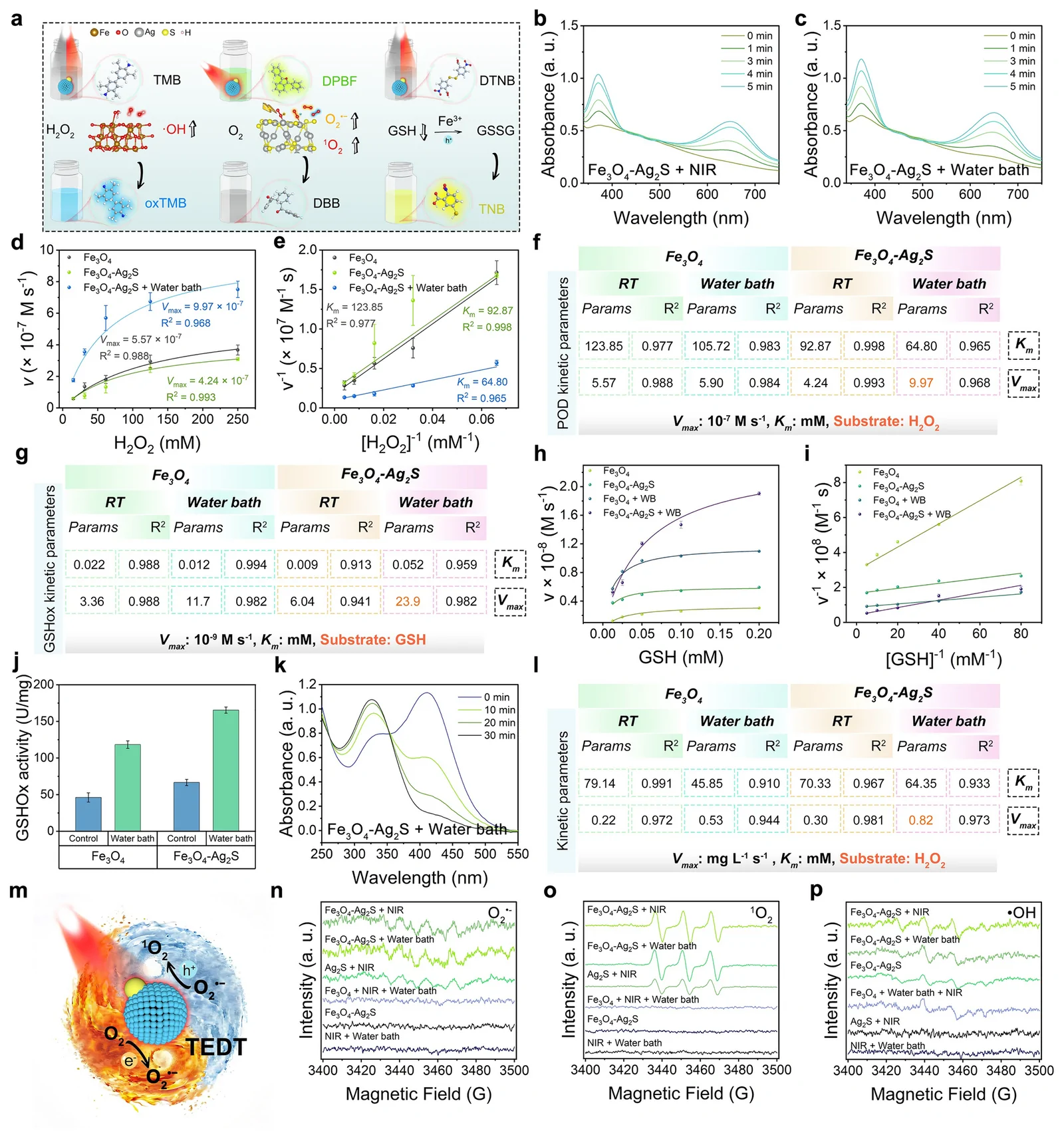
Analysis of enzyme–thermoelectric co-catalysis in solution environment. **a** Schematic for multi-enzyme catalytic activity detection. **b, c** TMB-based •OH generation (NIR/water bath). **d** Michaelis–Menten kinetic curves of POD-like enzyme kinetic and **e** Lineweaver–Burk plot. **f** Summary of POD-like enzyme kinetic parameters. **g** Summary of GSHox-like enzyme kinetic parameters. **h** Michaelis–Menten kinetic curves of GSHox-like enzyme kinetic and **i** Lineweaver–Burk plot. **j** DTNB indicated GSHox-like activity. **k** DTNB indicated GSH consumption of Fe_3_O_4_–Ag_2_S + water bath. **l** Summary of CAT-like enzyme kinetic parameters. **m** Schematic diagram of TEDT catalysis. ESR spectra of **n** O_2_^.^^−^, **o** ^1^O_2_, **p** •OH under different conditions. The NIR source was an 808 nm laser (0.4 W cm^−2^). The temperature of the water bath was at 60 °C. Data were presented as mean ± S. D. (*n* = 3)

Given the favorable photothermal properties of Fe_3_O_4_–Ag_2_S and its Fenton activity (Fig. 3a), we investigated its thermoelectric effect and enhanced enzyme-like activity. Since Fenton activity lacks in Ag_2_S [49], the •OH generation efficiencies of Fe_3_O_4_ and Fe_3_O_4_–Ag_2_S under various conditions were compared utilizing TMB as an indicator, as illustrated in Figs. 3b, c, and S7. The thermal effects induced by laser irradiation and water bath heating enhanced Fe_3_O_4_–Ag_2_S to produce •OH, surpassing Fe_3_O_4_ when both heat sources were applied simultaneously. This observation rules out mere thermal enhancement and confirmed the contribution of thermoelectric effects. Terephthalic acid detection further corroborated this trend (Fig. S8), suggesting that the thermoelectric effects enhanced POD-like activity in Fe_3_O_4_–Ag_2_S, while the minor improvement observed in Fe_3_O_4_ was thermally driven. To further validate these conclusions, the POD-like activities of Fe_3_O_4_–Ag_2_S and Fe_3_O_4_ were assessed. Kinetic curves were fitted using the Michaelis–Menten model and Lineweaver–Burk plots, as shown in Figs. 3d, e, and S9. The kinetic parameters summarized in Fig. 3f indicated that Fe_3_O_4_–Ag_2_S exhibited a 1.5-fold higher POD-like activity than Fe_3_O_4_ under thermoelectric conditions, with a reduced *K*_*m*_, confirming enhanced catalytic efficiency. The POD-like activity trends were further confirmed by guaiacol assay (Fig. S9c), consistent with the TMB results. The POD-like activity also demonstrated GSH depletion. 5,5'-Dithiobis-(2-nitrobenzoic acid) (DTNB) was used to detect GSH consumption. Similarly, DTNB was employed as a chromogenic substrate to investigate the GSHox-like enzymatic activity, and the corresponding kinetic data are summarized in Fig. 3g. As shown in Fig. 3h, l, the kinetic curves were fitted using the Michaelis–Menten model and Lineweaver–Burk plots. The results revealed that Fe_3_O_4_–Ag_2_S exhibited the highest GSHox-like enzymatic activity under water bath, further confirmed that the thermoelectric effect significantly enhanced the POD-like activity of Fe_3_O_4_–Ag_2_S. In addition, we calculated the enzyme catalytic activity unit under the same conditions, as shown in Fig. 3j, further verifying the above conclusion. As illustrated in Figs. 3k, and S11, Fe_3_O_4_–Ag_2_S showed well-characterized GSH depletion at room temperature, which was significantly accelerated by a water bath. This finding further substantiated the POD-like activity of Fe_3_O_4_–Ag_2_S and its enhancement via the thermoelectric effect.

In addition to its POD-like activity, Fe_3_O_4_ has been reported to catalyze the oxygen evolution of H_2_O_2_ [50, 51], a feature that helps mitigate hypoxia. Consequently, we investigated the oxygen evolution properties of both Fe_3_O_4_ and Fe_3_O_4_–Ag_2_S, as illustrated in Fig. S12a. The results indicated that Fe_3_O_4_–Ag_2_S demonstrated significant CAT-like activity, while the CAT-like activity of Fe_3_O_4_ was enhanced under a water bath and NIR irradiation. Notably, either water bath or NIR treatment alone induced a faster O_2_ generation rate in Fe_3_O_4_–Ag_2_S compared to dual treatment in Fe_3_O_4_. To further elucidate the oxygen generation rate, we analyzed the differentiated curves in Fig. S12b. The maximum O_2_ production rates for Fe_3_O_4_–Ag_2_S under NIR and water bath treatment reached 0.64 and 0.98 mg L^−1^ s^−1^, respectively, whereas the highest rate observed for dual-treated Fe_3_O_4_ was only 0.38 mg L^−1^ s^−1^. This phenomenon suggested that the enhancement is not solely due to thermal acceleration but is more likely attributed to thermoelectric effects.

Hole involvement in thermoelectric-enhanced catalysis was directly probed using AgNO_3_ as a hole quencher. The pronounced suppression of O_2_ generation and CAT-like activity upon AgNO_3_ introduction (Fig. S12a) confirmed that hole-mediated pathways are critical for the observed catalytic enhancement. To quantify the CAT-like enzymatic activity, the kinetic curves were fitted using the Michaelis–Menten model and Lineweaver–Burk plots, as shown in Fig. S12c, d. The summarized kinetic parameters are presented in Fig. 3l. The results indicate that Fe_3_O_4_–Ag_2_S subjected to water bath treatment exhibited the highest maximum catalytic reaction rate. Given that O_2_ serves as a common substrate in thermoelectric catalytic processes, these findings prompted us to further investigate the thermoelectrocatalytic performance of the Fe_3_O_4_–Ag_2_S nanoplatform. To distinguish heterojunction effects from simple coexistence, a physical mixture of Fe_3_O_4_ and Ag_2_S (denoted as Fe_3_O_4_ + Ag_2_S) was prepared as a control. Unlike the heterojunction-coupled Fe_3_O_4_–Ag_2_S composite, this mixture exhibited significantly lower catalytic activity, comparable to individual components (Fig. S10). The failure to reproduce the enhancement, despite identical composition, demonstrates that intimate interfacial coupling is essential for the observed enzyme-like catalytic activity.

As shown in Fig. 3m O_2 _^.^^−^ is commonly produced in thermoelectric catalytic processes. To detect O_2 _^.^^−^, DHR123 was employed as a fluorescent indicator. As illustrated in Fig. S13, no significant increase in fluorescence intensity was observed for either Fe_3_O_4_ or Fe_3_O_4_–Ag_2_S in a dark, room-temperature environment. Moreover, upon laser or water bath treatment, Fe_3_O_4_–Ag_2_S exhibited notable fluorescence emission, indicating the O_2 _^.^^−^ production. In contrast, Fe_3_O_4_ did not show any significant change under identical conditions. In addition, the effect of different water bath temperatures on O_2 _^.^^−^ generation was investigated, as shown in Fig. S13f, g. Compared with Fig. S13c, d, a larger temperature difference resulted in a higher yield of O_2 _^.^^−^. Furthermore, when superoxide dismutase (SOD) was introduced into the reaction system, as shown in Fig. S13i–l, the fluorescence signal weakened after the addition of exogenous SOD, confirming that the observed fluorescence enhancement phenomenon was indeed attributable to superoxide anions. This observation was consistent with the characteristics of thermoelectric catalytic activities, in which a greater temperature gradient induced a higher voltage, thereby leading to enhanced catalytic efficiency. These findings further confirmed that laser exposure or a water bath could effectively activate thermoelectrocatalytic activity. ^1^O_2_ is an oxidation product of O_2 _^.^^−^ and can be generated via holes or  •OH [52, 53]. Based on the thermoelectric properties and POD-like activity of Fe_3_O_4_–Ag_2_S, we examined ^1^O_2_ generation of Ag_2_S and Fe_3_O_4_–Ag_2_S under different conditions using 1,3-diphenylisobenzofuran (DPBF). As shown in Fig. S14, neither Ag_2_S nor Fe_3_O_4_–Ag_2_S exhibited DPBF degradation in a dark environment at room temperature. However, Fe_3_O_4_–Ag_2_S demonstrated significantly enhanced DPBF degradation under a water bath. This finding further substantiated the thermoelectrocatalytic properties of Fe_3_O_4_–Ag_2_S. ESR results (Fig. 3n–p) further confirmed thermoelectric-driven ROS generation and supported the thermoelectric enhancement of POD-like activity.

To elucidate the energy conversion process in thermoelectric catalysis, we systematically investigated the relationship between thermoelectric conversion and electrochemical performance. Using alternating hot and cold water baths to control the temperature gradient, we monitored the corresponding electrical responses. As shown in Fig. S15, the current exhibited periodic variations during heating/cooling cycles, indicating that Fe_3_O_4_–Ag_2_S nanoparticles efficiently generate electricity under a 15 °C temperature gradient. Both current and potential remained stable throughout multiple cycles, confirming robust thermoelectric conversion. Furthermore, larger temperature gradients produced significantly higher currents (Fig. S15b, c) and correspondingly increased ROS production. This positive correlation between temperature gradient, electrical output, and ROS yield demonstrates effective coupling between thermoelectric and electrochemical conversion processes. To verify the origin of ^1^O_2_, we performed ESR spectroscopy with selective hole/electron quenching and •OH modulation. The ^1^O_2_ signal nearly disappeared upon simultaneous quenching of electrons and holes (Fig. S16). H_2_O_2_ addition significantly increased ^1^O_2_ yield, while subsequent electron quenching reduced it, identifying O_2_^.^^−^ as the primary ^1^O_2_ precursor. Notably, Ag_2_S alone produced less ^1^O_2_ than Fe_3_O_4_–Ag_2_S under water bath (Fig. S14c, d), despite its higher thermoelectric component, suggesting that heterojunction formation enhances thermoelectrocatalytic properties.

### Energy Band Characterization and Molecular Orbitals Analysis

**Fig. 4:**
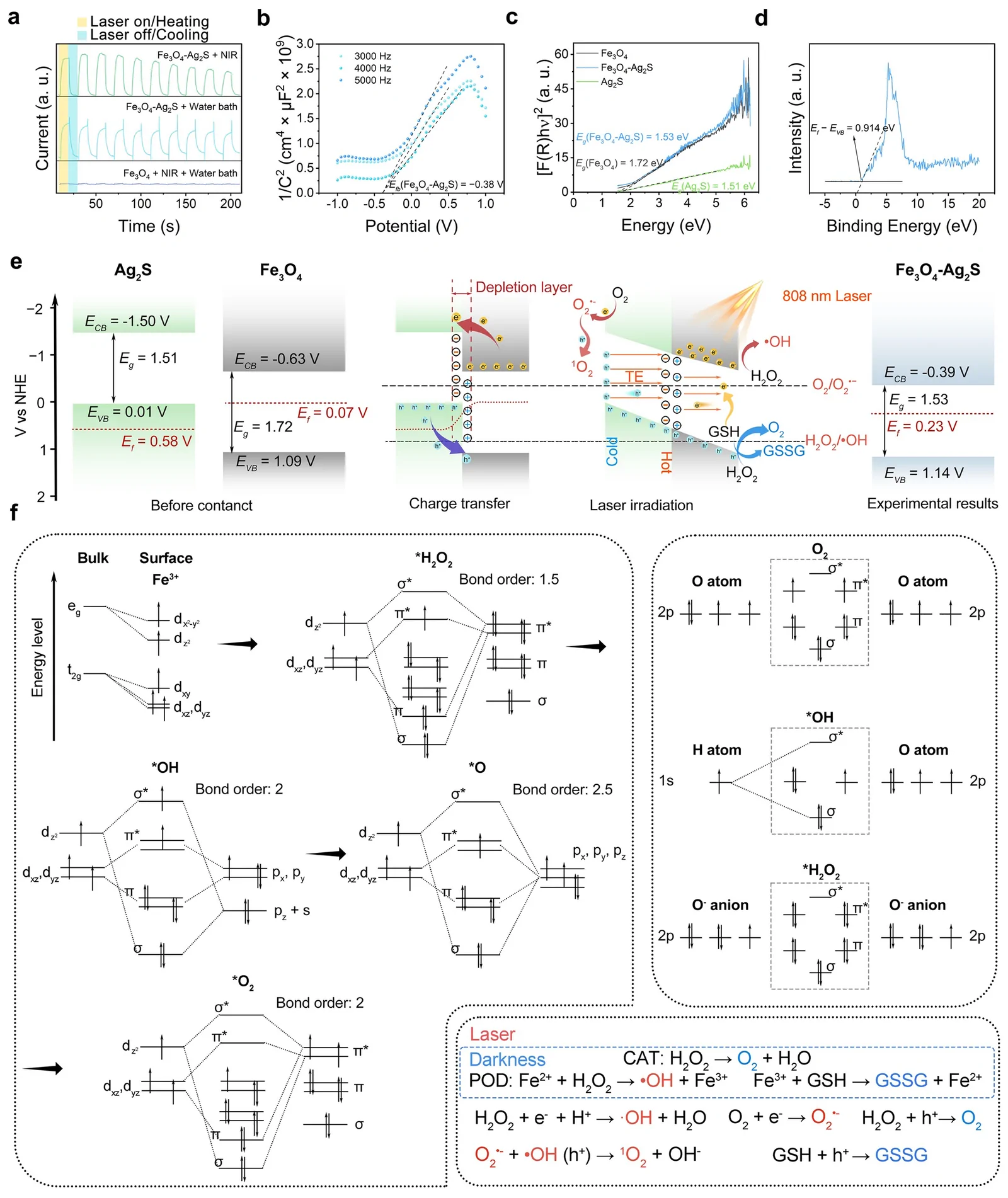
Energy band structure and enzyme–thermoelectric co-catalysis mechanism. **a** Photo-/thermoelectric current responses. **b** Mott–Schottky curves of Fe_3_O_4_–Ag_2_S. **c** UV–Vis–NIR diffuse reflectance spectra after Kubelka–Munk transformation. **d** XPS valence band spectra of Fe_3_O_4_–Ag_2_S. **e** Energy band alignment under NIR irradiation. **f** Molecular orbital theory analysis of oxygen evolution pathway

Based on these experimental findings, we analyzed thermoelectric effects and spin-related electronic modulation to elucidate the physical basis for the observed enhancement. These mechanistic insights are supported by structural, spectroscopic, and electronic evidence, and are presented as consistent interpretations that rationalize the experimental trends, rather than as fully resolved dynamic mechanisms. In light of the observed CAT- and POD-like activities, as well as the thermoelectric properties in solution, we conducted a further analysis of the energy band structure of Fe_3_O_4_–Ag_2_S. First, we investigated the thermal currents generated under laser irradiation or water bath heating to validate the presence of the thermoelectric effect. As illustrated in Fig. 4a, compared to Fe_3_O_4_, significant currents were recorded for Fe_3_O_4_–Ag_2_S upon application of either laser or water bath treatment to Fe_3_O_4_–Ag_2_S, thereby confirming the occurrence of the thermoelectric effect. To elucidate the mechanism underlying the enhancement of Fe_3_O_4_ enzyme catalysis by the thermoelectric effect of Ag_2_S, a detailed discussion of the energy band structure of Fe_3_O_4_–Ag_2_S was presented.

First, to determine the Fermi energy (*E*_*f*_) positions, the flat-band potentials of Ag_2_S, Fe_3_O_4_, and Fe_3_O_4_–Ag_2_S were obtained using the Mott–Schottky test. As illustrated in Figs. 4b and S17a, b, the negative slope observed for Ag_2_S confirmed that Ag_2_S is a *p*-type semiconductor, while both Fe_3_O_4_ and Fe_3_O_4_–Ag_2_S exhibited positive slopes, indicating typical *n*-type semiconductor behavior. Consequently, the in situ growth of Ag_2_S established a p–n junction structure. Furthermore, based on the Mott–Schottky diagram and the Nernst equation, the flat-band potentials (*E*_fb_ vs. NHE) for Ag_2_S, Fe_3_O_4_, and Fe_3_O_4_–Ag_2_S were determined to be 0.58, 0.07, and 0.23 V, respectively. These values are numerically equivalent to *E*_*f*_. When two semiconductors are in contact, their *E*_*f*_ values tend to equilibrate, resulting in the *E*_*f*_ of Fe_3_O_4_–Ag_2_S falling between those of Ag_2_S and Fe_3_O_4_. Subsequently, the optical bandgap (*E*_*g*_) was evaluated using UV–Vis diffuse reflectance spectroscopy. Following conversion via the Kubelka–Munk formula, as shown in Fig. 4c, the *E*_*g*_ values for Ag_2_S, Fe_3_O_4_, and Fe_3_O_4_–Ag_2_S were found to be 1.51, 1.72, and 1.53 eV, respectively. The observed narrowing of the *E*_*g*_ for Fe_3_O_4_–Ag_2_S was attributed to the modification by Ag_2_S.

Additionally, the energy difference between *E*_*f*_ and the valence band (*E*_VB_) was assessed using XPS valence band spectra, as illustrated in Figs. 4d and S17c, d. The energy differences for Ag_2_S, Fe_3_O_4_, and Fe_3_O_4_–Ag_2_S were determined to be − 0.57, 1.02, and 0.91 eV, respectively. Furthermore, valence band spectra were analyzed to obtain the experimental *d* band center values for Ag_2_S, Fe_3_O_4_, and Fe_3_O_4_–Ag_2_S, which were 2.051, 3.092, and 2.990 eV, respectively [54]. A lower *d* band center was more conducive to the adsorption of substrates and intermediates. Based on these results, an energy band diagram illustrating the heterojunction formation process was constructed. As depicted in Fig. 4e, upon contact between Ag_2_S and Fe_3_O_4_, a hole concentration gradient developed, directed from the *p*-type Ag_2_S to the *n*-type Fe_3_O_4_. This gradient resulted from the differing majority carrier types—holes in Ag_2_S and electrons in Fe_3_O_4_, leading to the diffusion of holes from Ag_2_S to Fe_3_O_4_. Conversely, due to the higher electron concentration in Fe_3_O_4_, electrons diffused from Fe_3_O_4_ to Ag_2_S. In Ag_2_S, the depletion of holes created a negatively charged layer resulting from uncompensated ionized acceptors. Similarly, a positively charged region developed on the Fe_3_O_4_ side, and the space charge layer progressively thickened as carriers diffused. The internal electric field within the space charge layer opposed further diffusion, eventually reaching equilibrium, as illustrated in Fig. 4e. Upon laser irradiation, the thermoelectric properties of Ag_2_S facilitated hole drift from the hot end to the cold end, generating a thermoelectric field directed toward the space charge layer, as shown in Fig. 4e. This thermoelectric field exerted a forward bias on the p–n junction, counteracting the built-in electric field. The thermoelectric field significantly altered the band bending within the barrier region, which had low carrier concentration and high resistance compared to the relatively high carrier concentration and low resistance in the Ag_2_S or Fe_3_O_4_ regions. Since the thermoelectric field opposed the built-in electric field, it reduced the electric field strength within the potential region, thereby decreasing the space charge. Consequently, both the width of the depletion region and the height of the potential barrier decreased.

These changes disrupted the original carrier balance in the p–n junction, causing diffusion exceeding drift. Hot electrons, excited by the 808 nm laser in Fe_3_O_4_, diffused from the Fe_3_O_4_ (*n* region) to the Ag_2_S (*p* region), while holes diffused in the opposite direction. This non-equilibrium injection of minority carriers facilitated various intriguing reactions under 808 nm laser activation, including electron-promoted oxygen reduction and hole-accelerated oxidation of H_2_O_2_ and GSH. These processes resulted in thermoelectric catalytic reactions and enhanced CAT and POD-like activities. When the laser was turned off, the thermoelectric field weakened or disappeared, causing the space charge layer to thicken and increasing the potential barrier in the junction region. This hindered carrier back-migration, and the drift current gradually increased until it equaled the diffusion current, ultimately restoring the p–n junction to a stable state.

To further elucidate the enhancement of CAT-like activity in high-spin Fe_3_O_4_, the bond order throughout the reaction process was analyzed using molecular orbital theory, as depicted in Fig. 4f. Bond order is defined as half the difference between the number of bonding and antibonding electrons. The molecular orbitals corresponding to reaction intermediates (O_2_, *OH, *H_2_O_2_) were schematically represented within the dashed box on the right. In contrast, the left dashed box illustrated the d electron configuration of Fe^3+^ at the catalytic surface. Previous studies had indicated that the stable spin configuration of the iron cation existed in a high-spin state [55]. Furthermore, to confirm the effect of Jahn–Teller distortion on the spin state of Fe^3+^, the spin states of Fe^3+^ in Fe_3_O_4_–Ag_2_S and Fe_3_O_4_ were detected using electron paramagnetic resonance (EPR) spectroscopy. As shown in Fig. S18, Fe_3_O_4_–Ag_2_S exhibited an EPR signal with higher intensity or modified features compared to Fe_3_O_4_, confirming that the Fe^3+^ sites in Fe_3_O_4_–Ag_2_S have *d* electrons with a higher-spin state. Although direct spin-state quantification by Mössbauer spectroscopy was not performed, multiple complementary results support heterojunction-induced spin-related electronic modulation. EPR spectra (Fig. S18) revealed clear changes in paramagnetic Fe-related centers after heterojunction formation, indicating modified local electronic and spin environments, variations widely recognized as sensitive probes of ligand field symmetry and spin structure in Fe-based oxides [56–58].

These EPR observations were fully consistent with the XPS Fe 2*p* spectral evolution, which revealed binding energy shifts and satellite feature changes indicative of modified ligand field strength and electronic configuration, as well as with Fe K-edge XANES/EXAFS results showing Fe–O bond distortion and reduced local symmetry. Together, these spectroscopic results demonstrated that heterojunction coupling induced lattice distortion and electronic reconstruction at Fe sites, provided a physically well-established pathway for modulating spin polarization and spin-dependent electronic states. Moreover, the observed structure–activity correlations, in which catalytic and photothermal performance systematically track the degree of lattice distortion and electronic modulation, further supported the involvement of spin-related electronic effects in governing the catalytic behavior. Specifically, this high-spin state of Fe^3+^ permitted each *t*_2*g*_ and *e*_*g*_ orbital to be occupied by an unpaired electron with a parallel spin, thereby positioning the entire *d* shell layer as a high-spin-state catalytic active center. Due to symmetry conservation, the contributions of the *d*_*x*_^*2*^_*−y*_^*2*^ and *d*_xy_ orbitals of the cations were considered negligible [59]. During catalysis, oxygen evolved through intermediates such as *H_2_O_2_, *OH, *O, and *O_2_ (where * denotes the active site). Higher bond order values indicated stronger orbital interactions between the cation and reacting intermediates. As shown in Fig. 4f, the bond order of the *O intermediate (2.5) exceeded that of the final *O_2_ intermediate (2.0), leading to *O_2_ release and continuation of catalytic cycle. Additionally, Fe^3+^ in the lowest spin state and Ag^+^ were analyzed, as shown in Fig. S17e. The bond order of each reaction intermediate was lower than in the high-spin state, indicating reduced stability and unfavorable conditions for CAT-like activity. This suggests that spin modulation enhanced CAT-like activity. A summary of laser-dependent responses was illustrated in Fig. 4f. Oxidation of H_2_O_2_ and GSH occurred in the dark via CAT and POD-like activities. Under 808-nm laser irradiation, O_2_ reduction and enhanced H_2_O_2_/GSH oxidation were promoted by thermoelectric and high-spin effects, achieving a synergistic catalytic effect between enzymatic and thermoelectric processes.

Although the conclusions drawn from the molecular orbital theory analysis align well with the experimental observations, previous studies had indicated that in pure Fe_3_O_4_ crystals, Fe^3+^ ions at the octahedral sites are Jahn–Teller inactive [60]. Although the surface-state octahedral sites had natural symmetry breaking, the weak Jahn–Teller distortion alone may not fully account for the enhanced catalytic performance. Consequently, the anomalous catalytic activity observed for Fe_3_O_4_–Ag_2_S required further clarification. Additionally, the notably higher ^1^O_2_ yield of Fe_3_O_4_–Ag_2_S remained for further study. Moreover, molecular orbital theory cannot predict whether the *O_2_ remains spin-polarized after oxidation. To further investigate these critical issues and validate our experimental findings at the theoretical level, DFT calculations were performed.

### DFT Calculations

**Fig. 5:**
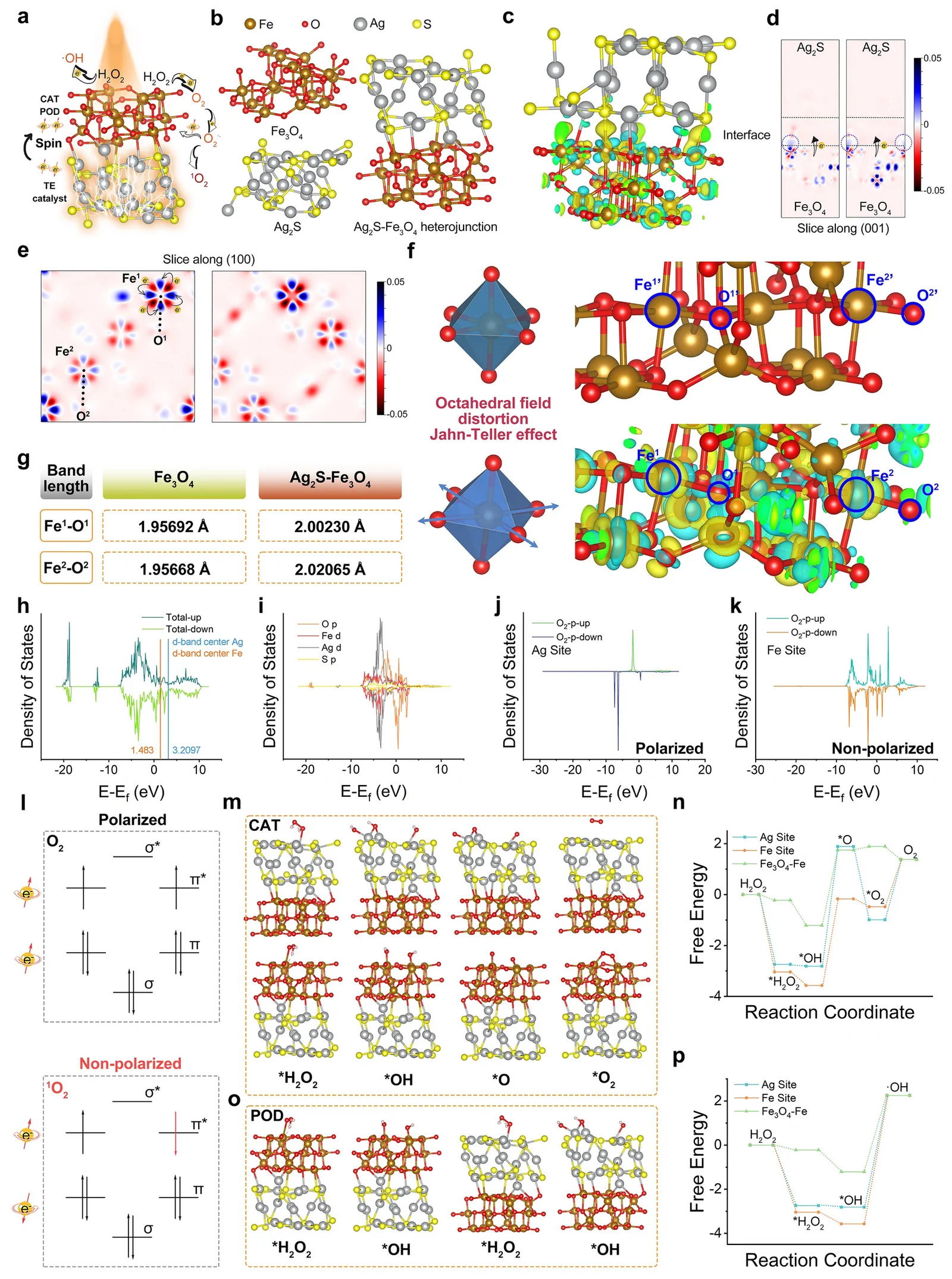
DFT calculations. **a** Spin-enhanced co-catalysis mechanism. **b** Optimized crystal models. **c** Differential charge densities of Fe_3_O_4_–Ag_2_S, and section plots along the **d** (001) and e (100) crystal plane. **f** Schematic illustration of the Jahn–Teller distortion and magnified view of the differential charge density at the heterojunction interface and the Fe_3_O_4_ matrix at the corresponding position. **g** Fe–O bond lengths labeled in **f**. **h** DOS plots of Fe_3_O_4_–Ag_2_S. The PDOS plots of **i** Ag, S, Fe, and O bonding orbitals, and **j–k** O_2_ adsorbed at Ag/Fe sites. **l** Molecular orbital illustration of the ^1^O_2_ and O_2_^.^^−^. **m–p** Simulated CAT/POD-like catalytic pathways and energy profiles

Figure 5a illustrates the spin-state-enhanced thermoelectric synergy for enzyme-mimetic catalysis. Firstly, the energies of different surface termination modes of Ag_2_S and Fe_3_O_4_ were evaluated to select the surface with the lowest energy for subsequent structural optimization. Four surfaces were considered for each material, and the results are shown in Fig. S19a, b. We chose the surface termination method 1 with the lowest energy as the starting model for the subsequent heterojunction structure optimization. Figure 5b presents the optimized atomic models of Ag_2_S, Fe_3_O_4_, and Fe_3_O_4_–Ag_2_S heterojunctions. To investigate interfacial charge transfer, charge density difference analysis was performed at the heterojunction interface and adjacent regions (Fig. 5c). The two-dimensional harge density difference map along the (001) plane (Fig. 5d) revealed that interfacial charge accumulation primarily originated from the *d*-orbitals of Fe_3_O_4_. Notably, significant electron redistribution within Fe_3_O_4_ was observed following heterojunction formation (Fig. 5c). To elucidate this phenomenon, differential charge density profiles along the (100) plane were analyzed (Fig. 5e). The electron cloud morphology associated with this charge redistribution corresponds to *d*_*x*_^*2*^_*−y*_^*2*^ and *d*_xy_ orbitals. Projected density of states (PDOS) analysis of Fe^1^–O^1^ bonding orbitals (Fig. S19c) confirmed strong hybridization between *d*_*x*_^*2*^_*−y*_^*2*^ and O 2*p* orbitals. As shown in Fig. 5e, *d*_xy_ electrons at octahedral Fe sites migrated to *d*_*x*_^*2*^_*−y*_^*2*^ orbitals, inducing electron accumulation in Fe–O bonds. This charge redistribution was likely a result of stress-induced crystal field distortions (Fig. 5f).

To validate this hypothesis, the Fe–O bond lengths in Fe_3_O_4_–Ag_2_S and pristine Fe_3_O_4_ were measured (Fig. 5f) and statistically compared (Fig. 5g). The elongated Fe–O bonds confirmed xy-plane crystal field distortions (tetragonal elongation), which drove charge redistribution. Furthermore, the variations in the average Fe–O bond lengths at the heterojunction interface were evaluated, including four octahedral and two tetrahedral sites. In standard Fe_3_O_4_, the average Fe–O bond lengths were approximately 2.067 Å for octahedral sites and about 1.877 Å for tetrahedral sites. In the interfacial layer of Fe_3_O_4_–Ag_2_S, the average Fe–O bond lengths at the octahedral sites were 1.997, 1.949, 1.956, and 1.997 Å, while those at the tetrahedral sites were 1.900 and 1.909 Å, respectively. The results revealed that the Fe–O bonds at the octahedral sites were contracted compared with the standard sample, whereas those at the tetrahedral sites were elongated. It should be noted that this change in the average bond length was not contradictory to the above analysis. The contraction of the average bond length arises from bond shortening along the *z*-direction. These findings were consistent with the fine structural features revealed by the FT-EXAFS analysis, further confirming the occurrence of the Jahn–Teller effect.

Consequently, the heterojunction-induced Jahn–Teller distortion generated energy splitting of *d*-orbitals, consistent with the theoretical model in Fig. 5j. These findings highlighted the critical role of heterostructures in promoting high-spin states within catalysts. During the tetragonal elongation process, the energy of the *d*_*x*_^*2*^_*−y*_^*2*^ orbital decreased, while the energy of the *d*_*z*_^*2*^ orbital increased due to the reduced bond length along the *z*-axis. As the substrates approached the central Fe ions along the *z*-axis, bonding with the *d*_*z*_^*2*^ orbital became more stable, favoring substrate adsorption. It is worth noting that the increased electron density in the Fe–O bonds within the xy-plane does not enhance bond energy. Instead, the increased *d*_*x*_^*2*^_*−y*_^*2*^ electron density exacerbates *d*–π* coupling, overfilling the antibonding orbitals, thereby reducing bond energy and increasing bond length.

To examine how Jahn–Teller distortion-induced high-spin states enhanced thermoelectric ^1^O_2_ production, density of states (DOS) analyses are conducted for the heterojunction and adsorbed systems. The closed bandgap in the total DOS (Fig. 5h) indicated superior charge transport properties of the heterojunction, facilitating thermoelectric charge transfer across interfaces. The positions of the *d* band centers of Fe and Ag, marked in Fig. 5h, showed a decreasing trend compared to pure Fe_3_O_4_ and Ag_2_S (Fig. S20) and were consistent with the *d* band center results from XPS valence band spectroscopy. This suggested that heterojunction formation facilitates the adsorption of substrates and intermediates. Orbital-resolved PDOS (Fig. 5i) demonstrated intense polarization in Fe–O and Ag–S bonding, with pronounced hybridization. Further PDOS analysis of O_2_ adsorbed at different sites (Fig. 5j, k) revealed distinct spin configurations. In thermoelectric catalysis, ^1^O_2_ typically formed via oxidation of O_2_^.^^−^ by holes or •OH, which differs from spin alignment of O_2_ in π* orbital (Fig. 5l).

Paramagnetic O_2_ exhibits asymmetric PDOS with net spin polarization, whereas antiferromagnetic ^1^O_2_ displays symmetric PDOS with spin depolarization. Figure 5j, k shows that O_2_ tends to be paramagnetic at Ag sites but antiferromagnetic at Fe sites, theoretically validating the heterojunction’s role in enhancing ^1^O_2_ yield. A comparative DOS analysis of O_2_ adsorbed on Fe_3_O_4_ further investigates electronic interactions. As illustrated in Fig. S21, the DOS profile of O_2_ on Fe_3_O_4_ exhibits moderate symmetry, suggesting that the high-spin state of Fe facilitates ^1^O_2_ generation. To quantitatively assess the symmetry of DOS profiles, the normalized asymmetry index was calculated for Figs. 5j, k, and S21 using the following formula:

10 $${\text{Asymmetry index}} = { }\frac{{\smallint \left[ {{\mathrm{DOS}} \uparrow \left( {\mathrm{E}} \right) + {\mathrm{DOS}} \downarrow \left( {\mathrm{E}} \right)} \right]}}{{\smallint {\mathrm{DOS}} \uparrow \left( {\mathrm{E}} \right) - \smallint {\mathrm{DOS}} \downarrow \left( {\mathrm{E}} \right)}} \times 100{{\% }}$$

where ∫DOS↑(E) and ∫DOS↓(E) represent the integrated areas of spin-up and spin-down density of states, respectively. The spin-resolved DOS was integrated within an energy range of [*E*_*f*_ − 10 eV, *E*_*f*_ + 10 eV], to capture the electronic states most relevant to thermally and carrier-driven catalytic processes. The calculated asymmetry indices for Figs. 5j, k, and S21 were 86.36%, 46.39%, and 59.16%, respectively. A higher asymmetry index indicated greater asymmetry. These results demonstrated pronounced symmetry in spin-polarized DOS, further corroborating the critical role of Jahn–Teller distortion in enhancing the spin asymmetry effect. The distinct symmetry variations among these configurations highlighted the structural sensitivity of spin polarization in Fe_3_O_4_-based systems. Importantly, within this framework, several experimental and theoretical observations suggest that spin-related electronic effects played a non-negligible role beyond conventional heterojunction charge separation alone. Spectroscopic evidence from XPS and Fe K-edge XANES/EXAFS revealed pronounced modifications in Fe coordination symmetry and ligand field strength, which were known to correlate with spin-dependent electronic structure in Fe-based systems. Consistently, DFT calculations shown redistribution of spin density and altered spin polarization at catalytically active sites upon defect–interface coupling, indicating that the reaction landscape was modified not only by band alignment or strain-induced band shifts. Together, these results supported the interpretation that spin-related electronic modulation contributed meaningfully though not exclusively to the observed catalytic kinetics.

Thermodynamic studies were conducted to elucidate enzymatic reaction mechanisms, which is widely used in many previous studies [20, 24, 61]. Intermediate states during CAT-like activity were simulated (Figs. 5m and S22), with relative energy profiles shown in Fig. 5n. Fe sites favor H_2_O_2_ adsorption and O_2_ desorption, optimizing the CAT-like reaction pathways. Similarly, POD-like intermediates were modeled (Figs. 5o and S22), and energy profiles (Fig. 5p) indicated Fe promotes H_2_O_2_ adsorption while Ag facilitated •OH desorption. Interestingly, as shown in Fig. 5n, p, Fe sites on pure Fe_3_O_4_ exhibited poor adsorption performance for H_2_O_2_ and •OH, resulting in suboptimal CAT and POD-like activity due to off-target interactions. As previously discussed in the charge density difference maps, the Jahn–Teller effect induced by heterojunction formation compressed bonds along the z-direction, enhancing substrate adsorption. These thermodynamic analyses confirmed that Jahn–Teller distortion-optimized Fe_3_O_4_ enhanced both CAT and POD-like reaction pathways by reducing energy barriers, thereby theoretically rationalizing the superior enzymatic performance of Fe_3_O_4_–Ag_2_S heterojunction.

### In Vitro Synergistic Antitumor Efficacy

**Fig. 6:**
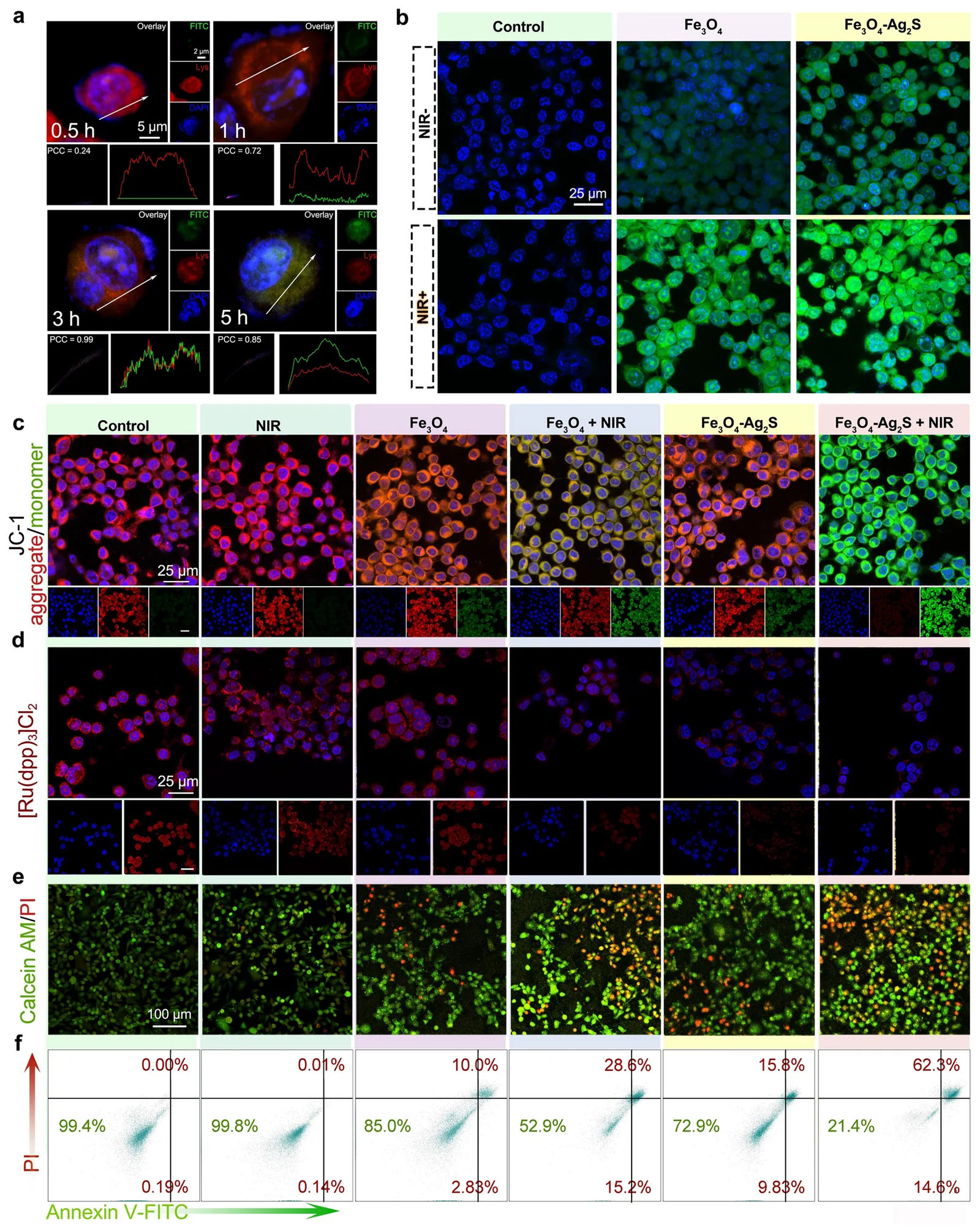
In vitro antitumor properties. **a** Time-dependent subcellular colocalization experiments. **b** ROS production in different treatment groups. **c** JC-1 indicated mitochondrial membrane potential changes in different treatment groups. **d** Intracellular O_2_ content indicated by [Ru(dpp)_3_]Cl_2_. **e** Live (AM)/ dead (PI) staining. **f** Apoptosis flow cytometry. The NIR light source was an 808 nm laser (0.4 W cm^−2^, 2 min)

Motivated by the promising catalytic therapeutic potential exhibited by Fe_3_O_4_–Ag_2_S heterostructures in simulated environments, we embarked on a comprehensive investigation to systematically unravel their antitumor activity. This was first achieved through a meticulous series of multimodal cellular-level evaluations. Firstly, to reveal the stability of the Jahn–Teller effect in a biological context, the spin state of Fe_3_O_4_–Ag_2_S after 48h of cultivation in serum-containing culture medium was detected using EPR spectroscopy, as shown in Fig. S18. The characteristic single-electron peak did not show any significant attenuation, confirming the stability of Fe_3_O_4_–Ag_2_S in vivo. In addition, multiple lines of indirect yet functionally relevant evidence supported the structural and electronic robustness of the heterojunction-induced lattice distortion following cellular internalization. Post-incubation HRTEM analyses as shown in Fig. S2f, g confirmed that the nanoparticles retain their crystalline framework and heterojunction morphology after exposure to biologically relevant media, indicating preserved structural integrity under physiological conditions. As an initial step, biocompatibility assessments were meticulously carried out utilizing methyl thiazolyl tetrazolium (MTT) assays, which involved the exposure of L929 fibroblasts and skeletal muscle myoblast cell line C2C12 to varying concentrations of Fe_3_O_4_–Ag_2_S nanoparticles. Notably, the results depicted in Fig. S23a, b reveals that even after a 24 h exposure to a relatively high concentration of 200 μg mL^−1^ Fe_3_O_4_–Ag_2_S nanoparticles, the cell viability remained impressively above 90%. This finding not only underscored the biosafety profile of Fe_3_O_4_–Ag_2_S but also fulfilled a critical prerequisite for their use in both in vivo and in vitro applications. Subsequent intracellular tracking studies employing FITC-labeled Fe_3_O_4_–Ag_2_S and Lyso-Tracker Red staining (Fig. 6a) revealed a dynamic lysosomal interaction pattern.

Quantitative colocalization analysis demonstrated time-dependent accumulation within lysosomal compartments, with Pearson's correlation coefficient (PCC) progressively increasing from 0.24 at 0.5 h to 0.99 at 3 h post-incubation. This rapid lysosomal entrapment aligned with typical endocytic pathways for nanoparticle internalization. Notably, the PCC decreased to 0.85 after 5h without significant FITC fluorescence attenuation, suggesting successful lysosomal escape, which is a crucial mechanism enabling cytoplasmic drug delivery and minimizing lysosomal degradation of therapeutic agents. Furthermore, as shown in Fig. S23c, colocalization analysis at 12 h post-phagocytosis demonstrated even more pronounced lysosomal escape, with PCC reduced to 0.75, and the intracellular FITC fluorescence intensity further increased, confirming our aforementioned analysis.

The therapeutic mechanism was further elucidated through ROS generation analysis using DCFH-DA. As illustrated in Fig. 6b, NIR irradiation of Fe_3_O_4_–Ag_2_S triggered an increase in intracellular ROS compared to controls. This pronounced ROS amplification established the photothermoelectric therapeutic potential of Fe_3_O_4_–Ag_2_S. Complementary mitochondrial membrane potential (ΔΨm) assessments using JC-1 fluorescence (Fig. 6c) revealed that Fe_3_O_4_–Ag_2_S + NIR treatment induced mitochondrial depolarization, as evidenced by an increase in the green/red fluorescence intensity ratio. This substantial ΔΨm collapse confirmed the activation of intrinsic apoptosis pathways, a critical determinant of therapeutic efficacy. To address tumor hypoxia limitations in photothermoelectric therapy, [Ru(dpp)_3_]Cl_2_-based oxygen detection (Fig. 6d) demonstrated that Fe_3_O_4_–Ag_2_S + NIR generated higher intracellular oxygen levels than baseline, attributed to the CAT-like activity of Fe_3_O_4_–Ag_2_S in decomposing endogenous H_2_O_2_. This oxygen self-supply mechanism created a positive feedback loop, enhancing ROS generation. Final therapeutic validation through MTT assays (Fig. S24a) and calcein AM/PI dual staining (Fig. 6e) showed that Fe_3_O_4_–Ag_2_S + NIR achieved 78.4% tumor cell death, significantly outperforming single-modality treatments. Flow cytometric apoptosis analysis (Fig. 6f) quantitatively confirmed a 76.9% apoptosis rate, demonstrating superior tumoricidal effects through synergistic photothermal–photodynamic–chemodynamic actions. In addition, to exclude the interference of simple thermal effects on the therapeutic outcome, we analyzed the cell viability after laser and water bath heating treatments for 0–5 min using the MTT assay. As shown in Fig. S24c, water bath treatment had only a slight impact on cell viability. Furthermore, according to the photothermal curve in Fig. S5a, at our therapeutic concentration, laser irradiation for 5 min raised the temperature from 25 to 45 °C in approximately 90s, and the temperature was maintained at 45 °C for about 3 min without significantly reducing cell viability.

To quantitatively assess the tumor cell–specific cytotoxicity of Fe_3_O_4_–Ag_2_S, we calculated the tumor cell/normal cell viability ratio, also referred to as the selectivity index (SI). A higher SI value indicates stronger selectivity toward killing tumor cells while sparing normal cells. At each nanoparticle concentration, the SI was determined using the following equation:

11 $${\mathrm{SI}} = \frac{{\text{Viability of Normal Cell}}}{{\text{Viability of Tumor Cell}}}$$

As shown in Fig. S24b, the SI values of Fe_3_O_4_–Ag_2_S were significantly greater than 1 across the normal cell lines tested. Notably, upon near-infrared irradiation, which activates its therapeutic functionality, the SI values increased markedly. This enhancement could be attributed to the selective destruction of a substantial fraction of 4T1 tumor cells through ROS generation or photothermal effects, while normal cells remained largely unaffected due to their lack of nanoparticle accumulation and potentially higher intrinsic antioxidant capacity. This high selectivity provided a basis for subsequent in vivo treatment. To verify the POD-like activity of the Fe_3_O_4_–Ag_2_S nanoplatform at the cellular level, its GSH depletion capacity was further investigated. First, we measured the total GSH content in 4T1 cells after various treatments. As shown in Fig. S25, the Fe_3_O_4_–Ag_2_S + NIR group led to the largest reduction in intracellular GSH levels. This confirmed that the hot holes generated by the thermoelectric effect, as proposed, effectively oxidize and deplete the key cellular antioxidant GSH, thereby disrupting the redox homeostasis. Second, to assess the downstream effects of GSH depletion, we performed glutathione peroxidase 4 (GPX4) immunofluorescence staining. As shown in Fig. S26, A marked decrease in GPX4 fluorescence intensity was observed specifically in the Fe_3_O_4_–Ag_2_S + NIR group. This provided direct cellular-level evidence that GSH depletion led to the inactivation of GPX4, a key enzyme that relies on GSH as a cofactor to reduce lipid peroxides.

### Multimodal Imaging and In Vivo Synergistic Antitumor Efficacy

Leveraging the synergistic X-ray attenuation properties of Ag combined with photothermal conversion capabilities of the heterojunction, the multimodal imaging performance of Fe_3_O_4_–Ag_2_S was systematically investigated. In vitro CT imaging studies (Fig. S27a, b) revealed significant contrast enhancement with concentration-dependent signal linearity. In vivo evaluations in 4T1 tumor-bearing mice demonstrated dual administration-dependent contrast kinetics: intratumoral injection (Fig. S27c–f) produced immediate signal amplification, while *i.v.* delivery (Fig. S27g) exhibited time-dependent tumor accumulation with optimal contrast observed at 12 h post-injection. Complementary PA imaging (Fig. S27h–j) demonstrated wavelength-specific signal enhancement, with PA signal at 808 nm showing concentration-dependent linearity, validating the material’s dual-modal CT/PA imaging capacity through plasmonic Ag_2_S and Fe_3_O_4_ interactions. Additionally, we calculated the signal-to-noise ratio (SNR) for the lowest concentration of Fe_3_O_4_–Ag_2_S, using the following formula:

12 $${\mathrm{SNR}} = 10 \times \log_{10} \frac{{A_{{{\mathrm{signal}}}} }}{{A_{{{\mathrm{noise}}}} }}$$

where *A*_signal_ and *A*_noise_ corresponding to the average gray values of the sample and background areas, respectively. The SNR value of the 500 μg mL^−1^ Fe_3_O_4_–Ag_2_S aqueous solution was calculated to be 5.41. The concentration at which the SNR exceeded a standard threshold for detectability of 3 was defined as the detection limit. Our analysis confirmed that the agent can be reliably detected at concentrations as low as 500 μg mL^−1^. Furthermore, we conducted a quantitative analysis of the CT signals at the tumor site, as shown in Fig. S28. We expanded the analysis of Fig. S27h by calculating the SNR using formula (12) for each concentration based on the PA signal intensity versus nanoparticle concentration. The SNR value at 62.5 μg mL^−1^ is 2.56, while that at 125 μg mL^−1^ is 5.27. Our analysis confirmed that the agent can be reliably detected at concentrations as low as 125 μg mL^−1^. The PA and CT imaging functions are expected to work well together and achieve multimodal imaging in vivo [62].

**Fig. 7:**
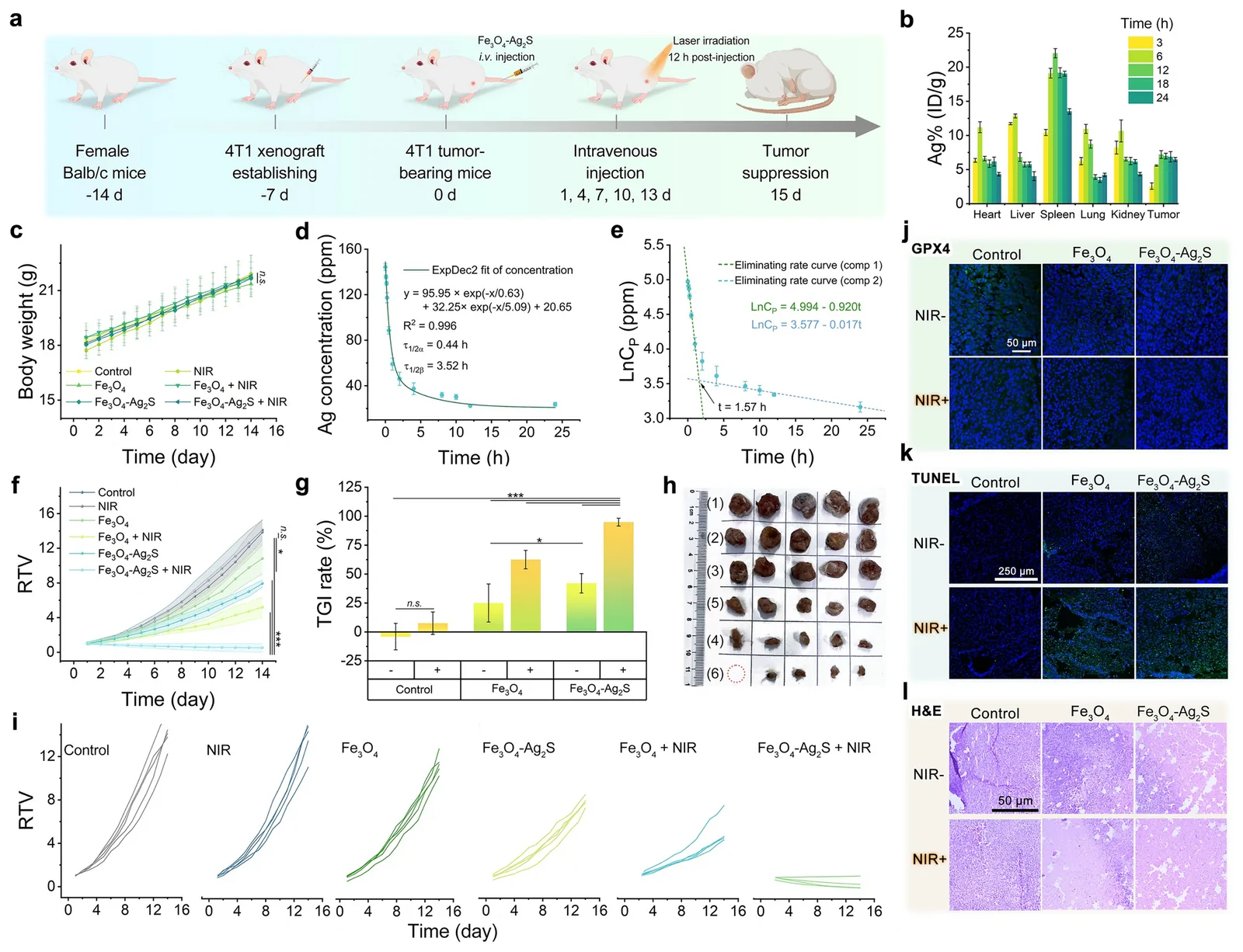
In vivo therapy against 4T1 tumor xenografts. **a** Treatment regimen in tumor-bearing mice. **b** ICP–OES indicated in vivo drug distribution. **c** Body weight curves. **b** Circulation profile and **e** elimination rate profile. **f** RTV curves. **g** Tumor inhibition rate. **h** Digital photographs of dissected tumors. **i** RTV curves of each mouse, **j** GPX4 immunofluorescent, **k** TUNEL staining, and **l** H&E-stained images. All data are represented as mean ± S. D., *n* = 5 (for Fig. 7c, f, and g) or 3 (for Fig. 7b, d, and e), *n. s.*: no significance *p < 0.05 **p < 0.01, ***p < 0.001

Leveraging these imaging-guided pharmacokinetic insights, therapeutic efficacy was evaluated in six cohorts of Balb/c mouse (Fig. 7a). Inductively coupled plasma optical (ICP–OES) biodistribution profiling (Fig. 7b) identified 12h post-*i.v.* injection as the optimal therapeutic window. Furthermore, given the well-documented cytotoxicity of Ag^+^ ions, to ensure the biosafety of Fe_3_O_4_–Ag_2_S, a quantitative assessment of the kinetics of Ag^+^ ion release from the Fe_3_O_4_–Ag_2_S was performed under simulated tumor microenvironmental conditions (pH 6.5, with 100 μM H_2_O_2_ and 100 μM GSH). The Ag⁺ concentration in the supernatant was meticulously measured using inductively coupled plasma mass spectrometry (ICP–MS) at various time points. As shown in Fig. S29a, the release of cytotoxic Ag⁺ ions were negligible, allowing us to confidently proceed with subsequent tumor treatment protocols. The Fe signal, in contrast, suffered from high baseline interference from the physiological background, which significantly reduced detection accuracy in blood and major organs, as shown in Fig. S29b.

Given that the above in vivo distribution experiments indicated that a large amount of Fe_3_O_4_–Ag_2_S was captured by the reticuloendothelial system such as the liver and kidneys in the early stage, we conducted the MTT experiments with HL-7702 hepatocytes, HK-2 renal cells to confirm the material's biocompatibility with the liver and kidneys, respectively. As shown in Fig. S29c, d, at high concentrations (200 μg mL^−1^), the hepatorenal cytotoxicity of Fe_3_O_4_–Ag_2_S in mice was disregarded. Even after intravenous injection and substantial accumulation in liver and kidney tissues, its excellent biocompatibility ensures low side effects. Importantly, the absolute Ag accumulation levels in the liver and kidney remained at low levels (Fig. S29e), consistent with the minimal Ag^+^ release detected by leaching-based ICP–MS analysis and supported the low cytotoxicity observed in hepatic and renal cell models, and further confirmed the absence of significant hepatotoxicity or nephrotoxicity under the applied dosage and treatment conditions. Furthermore, the accumulation of Ag^+^ in various major organs after different injection times of Fe_3_O_4_–Ag_2_S was detected. As shown in Fig. S29e, the amount of Ag^+^ accumulated during a treatment cycle was almost negligible, further demonstrating the excellent biocompatibility of Fe_3_O_4_–Ag_2_S. Body weight trajectories (Fig. 7c) confirmed treatment safety, with only minor fluctuations across groups. Pharmacokinetic modeling (Fig. 7d, e) revealed biphasic elimination: rapid *α*-phase clearance followed by prolonged *β*-phase retention, suggesting stable tumor deposition. To demonstrate that the treatment was properly activated in vivo, we assessed photothermal assess of the tumor-bearing mice during NIR irradiation. As shown in Fig. S30, the tumor region in the Fe_3_O_4_–Ag_2_S + NIR group exhibited a rapid and localized temperature increase, reaching a plateau of ~ 45 °C. It confirms the effective photothermal conversion of the nanoparticles at the tumor site. The 808 nm NIR irradiation employed in this work lied within the biological window and enabled sufficient light penetration for photothermal activation in small-animal tumor models. The average tumor size at the onset of treatment was ≈4 mm in diameter, which was comparable to the reported penetration depth of NIR-I light, allowing effective intratumoral heating [63–65]. After 14 days treatment, the tumor-bearing mice were killed and dissected. The tumors were removed and the relative tumor volume and inhibition rate were calculated. As shown in Fig. 7f–i, the Fe_3_O_4_–Ag_2_S + NIR group demonstrated exceptional tumor suppression with a tumor inhibition rate of 95%, outperforming Fe_3_O_4_ + NIR, attributed to Ag_2_S-mediated TEDT and Fe^3+^-driven enzyme-mimetic catalytic therapy. The tumor inhibition efficiency of Fe_3_O_4_–Ag_2_S was further compared with recently reported iron-based nanotherapeutic platforms. As shown in Table S2, Fe_3_O_4_–Ag_2_S exhibited higher efficacy under NIR stimulation than many recently published iron-based nanoplatforms.

To further elucidate the mechanism underlying the therapeutic effect, firstly, we assessed an in vivo ROS generation by DCFH-DA staining on tumor sections. As shown in Fig. S31a, a massive burst of green fluorescence exclusively in the Fe_3_O_4_–Ag_2_S + NIR group. This provided direct visual evidence that our therapy triggers oxidative stress response within the tumor tissue, corroborating our in vitro ROS findings and establishing a direct link to the proposed catalytic mechanism. Subsequently, immunofluorescence analysis (Fig. 7j) revealed glutathione peroxidase 4 (GPX4) inactivation due to Fe^3+^-catalyzed GSH depletion, inducing redox homeostasis disruption. Terminal deoxynucleotidyl transferase-mediated nick end labeling (TUNEL) assays (Fig. 7k) demonstrated extensive apoptosis with characteristic nuclear fragmentation, corroborated by hematoxylin and eosin (H&E)-stained necrotic zones (Fig. 7l). In addition, we performed Ki67 staining, a well-established marker for proliferating cells. As shown in Fig. S31b, a marked reduction in Ki67-positive cells was observed in the Fe_3_O_4_–Ag_2_S + NIR group compared to other groups. This critical data showed that our treatment not only kills existing tumor cells, but also potently suppressed the proliferative capacity of the surviving tumor cells. To further corroborate the alleviation of tumor hypoxia, hypoxia inducible factor-1α (HIF-1α) immunohistochemical (IHC) staining was performed on tumor sections after different treatments. HIF-1α was a key hypoxia-responsive transcription factor and served as a direct molecular indicator of intratumoral hypoxic status. As shown in Fig. S31c, the Fe_3_O_4_–Ag_2_S treated tumors exhibited markedly reduced HIF-1α expression compared with control groups, which was fully consistent with the [Ru(dpp)_3_]Cl_2_ results. This molecular-level evidence further confirmed that Fe_3_O_4_–Ag_2_S treatment effectively alleviated tumor hypoxia. Systemic biocompatibility was evidenced by preserved organ architecture in the heart, liver, spleen, lung, and kidney (Fig. S32). The long-term in vivo biosafety of Fe_3_O_4_–Ag_2_S was a critical consideration for its potential biomedical application. In addition to the acute and short-term safety indicators discussed above, long-term tolerance was further evaluated by survival analysis following systemic administration. As shown in Fig. S33, the survival rate of the treatment group was significantly higher than that of the control group, due to the therapeutic effect on the tumors. This observation was consistent with the stable body weight profiles and the histopathological examination of major organs, which revealed no evident tissue damage or pathological abnormalities under the applied dosage and treatment regimen. Taken together, these complementary in vivo assessments consistently demonstrated minimal long-term systemic toxicity and favorable biocompatibility of Fe_3_O_4_–Ag_2_S within the experimental timeframe of this study. Looking forward, extended long-term toxicity studies beyond the current experimental timeframe would be valuable to further evaluate any potential chronic effects. Regarding the degradation products of Ag_2_S, it was noteworthy that sulfide species (S^2−^/HS^−^) in biological systems was typically rapidly metabolized and oxidized to more stable and less toxic sulfur species, such as thiosulfate and sulfate, through endogenous enzymatic pathways [66, 67]. This physiological sulfur metabolism was expected to mitigate potential risks associated with trace sulfide species arising from Ag_2_S degradation.

## Conclusions

In summary, the Fe_3_O_4_–Ag_2_S heterojunction system, engineered via spin-state and Jahn–Teller optimization and p–n junction design, achieves synergistic tumor therapy through photothermal–thermoelectric coupling and multi-enzyme catalysis. The unidirectional carrier transport at the heterojunction interface minimizes electron–hole recombination, while the photothermal effect activates thermoelectric response, generating robust thermoelectric fields. Spin catalysis effects and Jahn–Teller distortion of high-spin Fe^3+^ enhance CAT-like H_2_O_2_/O_2_ conversion, alleviating tumor hypoxia, while thermoelectric on Ag_2_S nanoparticles catalyze O_2_ reduction to O_2 _^.^^−^, amplifying TEDT efficacy. The hot holes oxidize O_2 _^.^^−^ to ^1^O_2_. Concurrently, hot holes deplete GSH via oxidation, and Fe_3_O_4_-derived electrons boost POD-like  •OH generation, creating a self-reinforcing ROS cascade. In vivo studies validate a 95% tumor inhibition rate under NIR irradiation, significantly outperforming Fe_3_O_4_ alone (38%), calculated by tumor volume. The system’s dual-modal CT/PA imaging capabilities, driven by Ag and Fe’s X-ray attenuation and photothermal conversion, enable real-time tumor monitoring. DFT calculations further elucidate heterojunction-induced Jahn–Teller distortions, which optimize d-orbital splitting and spin-polarized carrier dynamics for enhanced catalytic activity. This work not only addresses critical challenges in TEDT—hypoxia and carrier recombination—but also pioneers a spin-engineered heterojunction platform for imaging-guided, enzyme-augmented cancer therapy, offering broad implications for nanodynamic and theranostic applications.

## Supplementary Information

Below is the link to the electronic supplementary material.

Supplementary file1 (DOCX 6882 KB)
