# Supplementary material for: Enhanced Spin-Engineering Photothermoelectric–Enzymatic Catalysis System via Lattice Mismatch-Induced Jahn–Teller Distortion for Tumor Therapy
Source: Nanomicro Lett. 2026 Apr 9;18:323. doi: 10.1007/s40820-026-02175-y (PMC13062157; doi:10.1007/s40820-026-02175-y)
Supplement: Supplementary file 1 — Supplementary file1 (DOCX 6882 KB) [file 40820_2026_2175_MOESM1_ESM.docx]

Supporting Information for

**Enhanced Spin-Engineering Photothermoelectric-Enzymatic Catalysis System *via* Lattice Mismatch-Induced Jahn-Teller Distortion for Tumor Therapy**

Pengyu Zang^1^, Meiqi Yang^1^, Chenghao Yu^1^, Rui Zhang^1^, Avez Sharipov^2,^ *, Ruifang Shen^3^, Dan Yang^1,^ *, Shili Gai^1,^ *, and Piaoping Yang^1,^ *

^1^Key Laboratory of Superlight Materials and Surface Technology, Ministry of Education, College of Materials Science and Chemical Engineering, Harbin Engineering University, Harbin 150001, P. R. China

^2^Tashkent Pharmaceutical Institute, Ministry of the Health of Uzbekistan, Tashkent 100015, Republic of Uzbekistan

^3^Laboratory for Space Environment and Physical Sciences, Harbin Institute of Technology, Harbin 150006, P. R. China

* Corresponding authors. E-mail: [sharipov.avez@gmail.com](mailto:sharipov.avez@gmail.com) (Avez Sharipov); [yangdan@hrbeu.edu.cn](mailto:yangdan@hrbeu.edu.cn) (Dan Yang); [gaishili@hrbeu.edu.cn](mailto:gaishili@hrbeu.edu.cn) (Shili Gai); [yangpiaoping@hrbeu.edu.cn](mailto:yangpiaoping@hrbeu.edu.cn) (Piaoping Yang)

**S1 Experimental Sections**

**S1.1 Materials**

Ferric chloride hexahydrate (FeCl_3_·6H_2_O, AR), sodium citrate (C_6_H_5_Na_3_O_7_, AR), sodium acetate (C_7_H_6_NNaO_2_, AR), silver nitrate (Ag(NO)_3_, AR), sodium sulfide nonahydrate (Na_2_S·9H_2_O, AR), ethylene glycol (C_2_H_6_O_2_, AR), ammonia solution (H_5_NO, AR, 25-28%) dihydrorhodamine 123 (DHR123, 95%), 5,5-dimethyl-1- pyrroline N-oxide (DMPO, 97%), sodium sulfate anhydrous (Na_2_SO_4_, 99%) and p-phthalic acid (TA, 99%) were purchased from Rhawn (Shanghai, China). Ethylenediaminetetraacetic acid disodium salt (C_10_H_14_N_2_Na_2_O_8_, AR), 1,3-diphenylisobenzofuran (DPBF, 97%), 5',5'-dithiobis (2-nitrobenzoic acid) (DTNB, 95%) and 2,2′,6,6′-tetramethylpiperidine (TEMP, 95%), were purchased from Sigma-Aldrich (Shanghai, China). Glutathione (reduced, 98%), 3,3',5,5'- tetramethylbenzidine (TMB, >98%), 2,7-dichlorofluorescein diacetate (DCFH-DA, 99%), thiazolyl blue tetrazolium bromide (MTT, 98%), Calcein-AM (AM, 99%), and propidium iodide (PI, 94%) were obtained from Aladdin (Shanghai, China). Phosphate-buffered saline (PBS), trypsin cell digestive juices, and RPMI-1640 were obtained from Procell (Wuhan, China). The cell hypoxia detection kit Ru(dpp)_3_Cl_2_- red fluorescence, JC-1 staining kit, and Hoechst 33342 were obtained from Beyotime Inst. Biotech. (Haimen, China). Annexin V-FITC/PI apoptosis detection kit was taken from Tianjin Sungene Biotech Co., Ltd. (Tianjin, China). FITC-labeled Goat Anti-Rabbit IgG and HSP60 Rabbit Anti-mouse antibody were purchased from Shenyang Wanlei Biotechnology Co., Ltd. (Liaoning China). The TdT-mediated dUTP nick-end labeling (TUNEL) cell apoptosis detection kit was also bought from Dalian Meilun Biotechnology Co., Ltd. Female Balb/c mice (4 weeks old) were purchased from Liaoning Changsheng Biotechnology Co., Ltd. (Liaoning, China). All chemical agents were directly used without any further purification. The Animal experiment was conducted with the approval of ethics by the Ethics Committee of the Second Affiliated Hospital of Harbin Medical University. Animal experiments were performed according to the Guidelines for the Care and Use of Laboratory Animals of the Drug Safety Evaluation Center of Harbin Medical University. Female Balb/c mice were subcutaneously transplanted with 4T1 cancer cells (100 µL) at 5 weeks of age and used for subsequent experiments when the tumor volume approached 80 mm^3^. Animal Experiments Ethical Approval: All animal experiments were approved by the Ethics Committee of the Second Affiliated Hospital of Harbin Medical University (Harbin, China), and performed according to the Guidelines for Care and Use of Laboratory Animals of the Drug Safety Evaluation Center of Harbin Medical University.

**S1.2 Experimental Apparatus**

The transmission electron microscope (TEM) graph was obtained from the FEI Tecnai T20 transmission electron microscope. X-ray diffraction (XRD) was measured by a Rigaku DMAX-2400 X-ray diffractometer equipped with Cu Kα radiation (*λ* = 0.154 nm) at 40 kV and 40 mA. X-ray photoelectron spectroscopy (XPS) was measured by Thermo Fisher Scientific ESCALAB 250XI. inductively coupled plasma-optical emission spectrometry (ICP-OES) was measured by Agilent Technologies 725. UV–vis absorption spectrum was measured by UV1601 spectrophotometer. UV–visible–near-infrared absorption spectrum was tested by Shimadzu UV3600. Dissolved oxygen content analysis from portable dissolved oxygen analyzer (JPBJ-608, Anhui Leici Instrument Co. Ltd.) The ESR spectra were obtained with Bruker EMX1598 spectrometer. Thermal images were recorded using an Inf Rec R300SR-HD infrared thermal imager. The flow cytometry S3 assays were conducted on a BD Accuri C6 flow cytometer (USA). The PA images were obtained using the Vevo LAZR-X system. A confocal laser scanning microscope (CLSM, Leica TCS SP8) was used to obtain the fluorescence image. In vitro and in vivo CT imaging experiments were performed on a small animal X-ray CT imaging system (Quantum GX, PerkinElmer). X-ray absorption fine structure (XAFS) spectroscopy was carried out using the *RapidXAFS* 2M (Anhui Absorption Spectroscopy Analysis Instrument Co., Ltd.) by transmission mode at 15 kV and 20 mA, and the Ge (620) spherically bent crystal analyzer with a radius of curvature of 500 mm was used for Fe.

**S1.3 Supplemental Notes**

It should be noted that the observed catalytic enhancement in this study could not be attributed to a single factor alone. Interfacial charge separation at the heterojunction, lattice-strain–induced electronic structure modulation, and spin-related electronic effects were expected to act concurrently and synergistically in the Fe_3_O_4_-Ag_2_S system. In this work, spin polarization was therefore not proposed as the sole origin of the enhanced catalytic activity, but rather as an important contributing factor that operates in concert with interfacial charge transfer and strain effects. Above all, the term “spin engineering” in this work was used to describe EPR-supported and lattice distortion–driven modulation of spin-related electronic structure, rather than a directly measured spin-state transition. The absence of Mössbauer spectroscopy and detailed magnetic measurements is explicitly acknowledged as a limitation, and such quantitative spin-state probes would be pursued in future studies.

The present DFT analysis, based on a bulk-derived (001) interface model, provides fundamental insights into the heterojunction-induced Jahn-Teller distortion and its electronic consequences. In practical nanoparticle systems, exposed faces and finite curvature can further modulate the interfacial lattice mismatch and strain distribution, potentially enhancing or spatially varying the degree of local distortion. Such nanoscale structural heterogeneity may introduce additional complexity to the lattice–electronic–spin coupling proposed herein. While a quantitative evaluation of the Jahn-Teller stabilization energy would offer a more rigorous metric for distortion strength, its reliable calculation for realistic, multi-faceted heterojunction nanoparticles requires extended models and remains a valuable direction for future theoretical work. Besides, the present DFT calculations were not intended to explicitly simulate thermoelectric carrier injection or non-equilibrium reaction energy barriers. The thermoelectric effect was intrinsically a non-equilibrium phenomenon driven by temperature gradients and carrier redistribution, whereas conventional ground-state DFT calculations were equilibrium methods that could not directly incorporate Seebeck-induce internal electric fields or thermally drive chemical potential gradients. Instead, the role of DFT in this work was to elucidate the intrinsic electronic-structure modulation induced by defect–interface coupling, including charge density redistribution, density-of-states evolution, and adsorption energetics, which established the necessary electronic-structure basis for carrier-mediated surface reactions. Consistent with prior studies on electric-field-assisted and thermoelectric-enhanced catalysis, the dynamic carrier-driven effects were primarily inferred from experimental evidence such as thermoelectrically induced voltage generation, enhanced carrier transport, and temperature/light-dependent catalytic enhancement rather than from explicit non-equilibrium reaction barrier calculations.

In the multimodal imaging section, beyond demonstrating the feasibility of CT and PA imaging individually, their complementary diagnostic value should be further highlighted. CT imaging provided high-resolution anatomical and density-based contrast, enabling accurate tumor localization and macroscopic assessment of nanoparticle accumulation, while PA imaging offered functional contrast based on optical absorption and photothermal conversion, allowing real-time monitoring of nanoparticle distribution and photothermal response under irradiation. By integrating CT and PA imaging, the system combined structural and functional information, facilitating accurate tumor targeting, confirmation of nanoparticle accumulation, and optimization of irradiation timing and region, thereby enabling more precise and reliable guidance for subsequent therapy.

As for the biodistribution analysis, Ag was selected as the quantitative tracer element for biodistribution analysis because its signal could be more accurately distinguished and quantified by ICP–OES due to the low background interference from endogenous biological elements.

In the *in vivo* therapeutic efficiency analysis section, the *in vivo* photothermal experiments demonstrated efficient temperature elevation throughout the tumor region. Even beyond macroscopic heating, localized photothermal conversion at the nanoparticle and cellular levels could generate steep micro- to nanoscale temperature gradients, which were sufficient to induce thermoelectric carrier redistribution and contributed to the observed catalytic and therapeutic effects.

**S2 Supplementary Tables and Figures**

**Table S1** Quantitative EXAFS Fitting Parameters for Fe K-edge in Fe_3_O_4_ and Fe_3_O_4_–Ag_2_S

| Sample | | Bond Type | R | | N | | σ^2^ | ΔE_0_ |  |
| --- | --- | --- | --- | --- | --- | --- | --- | --- | --- |
| Fe_3_O_4_ | | Fe-O Tat | 1.51 | | 3.9 | | 0.006 | -4.2 |  |
| Fe_3_O_4_ | | Fe-O Oct | 2.62 | | 5.8 | | 0.009 | -4.9 |  |
| Fe_3_O_4_–Ag_2_S | | Fe-O Tat | 1.60 | | 3.5 | | 0.015 | -3.5 |  |
| Fe_3_O_4_–Ag_2_S | | Fe-O Oct | 2.55 | | 6.3 | | 0.019 | -3.1 |  |
| **Table S2** The tumor growth inhibition rates (TGI) of other Fe-based nanomaterials | | | | | | | | |  |
| **No.** | **Nanoplatform** | | | **TGI** | | **Refs.** | | | |
| 1 | TpyFe(II)-FF-AM-cRGD | | | 64.3% | | Adv. Funct. Mater. **2025**, 2505343 | | | |
| 2 | GA-Fe-GOX-HA | | | 66.3% | | Adv. Compos. Hybrid. Mater. **2025**, 8, 338 | | | |
| 3 | Fe-DMOS | | | 89.1 % | | Adv. Mater. **2025**, e10010 | | | |
| 4 | HA/FeMo_DA_-LPs | | | 93.7% | | Angew. Chem. Int. Ed. **2025**, 64, e202511471 | | | |
| 5 | MAFe_3_O_4_ | | | 67% | | ACS Nano **2025**, 19, 21468–21483 | | | |
| 6 | PDA–Fe(III)–FA | | | 86.6% | | ACS Nano **2025**, 19, 28554–28575 | | | |
| 7 | Fe-TCPP(Cu)-HA | | | 76.65% | | Adv. Funct. Mater. **2025**, e02635 | | | |
| 8 | NP_siCD47_@Fe-TA | | | 74% | | Adv. Funct. Mater. **2025**, 35, 2417548 | | | |
| 9 | MNRs@GOx/Cu/HSA | | | 97% | | ACS Nano **2025**, 19, 15040–15054 | | | |
| 10 | FeS-CRISPR/Cas9 | | | 69% | | ACS Nano **2025**, 19, 18188–18202 | | | |
| 11 | Fe–N_5_ | | | 90% | | J. Am. Chem. Soc. **2025**, 147, 17372–17383 | | | |
| 12 | FeCoCN | | | 94% | | Adv. Funct. Mater. **2025**, 35, 2423783 | | | |
| 13 | Zr-Fe MOF@Ribociclib@Herceptin | | | 66.66% | | Adv. Funct. Mater., **2025**, 2508310 | | | |
| 14 | ZnO_2_@PDA-Fe | | | 98.92% | | Adv. Mater. **2024**, 36, 2412227. | | | |
| 15 | Fe_1_Mn_1_–NC_e_ | | | 95.74% | | J. Am. Chem. Soc. **2024**, 146, 10023–10031 | | | |
| 16 | Fe_3_O_4_-αPD-L1 | | | 90% | | J. Am. Chem. Soc. **2024**, 146, 22455–22468 | | | |
| 17 | Au–Fe_2_C | | | ≈90% | | Adv. Mater. **2024**, 36, 2307006 | | | |
| 18 | FeP@HCPT-HA | | | 80.5% | | Adv. Funct. Mater. **2024**, 34, 2313540 | | | |


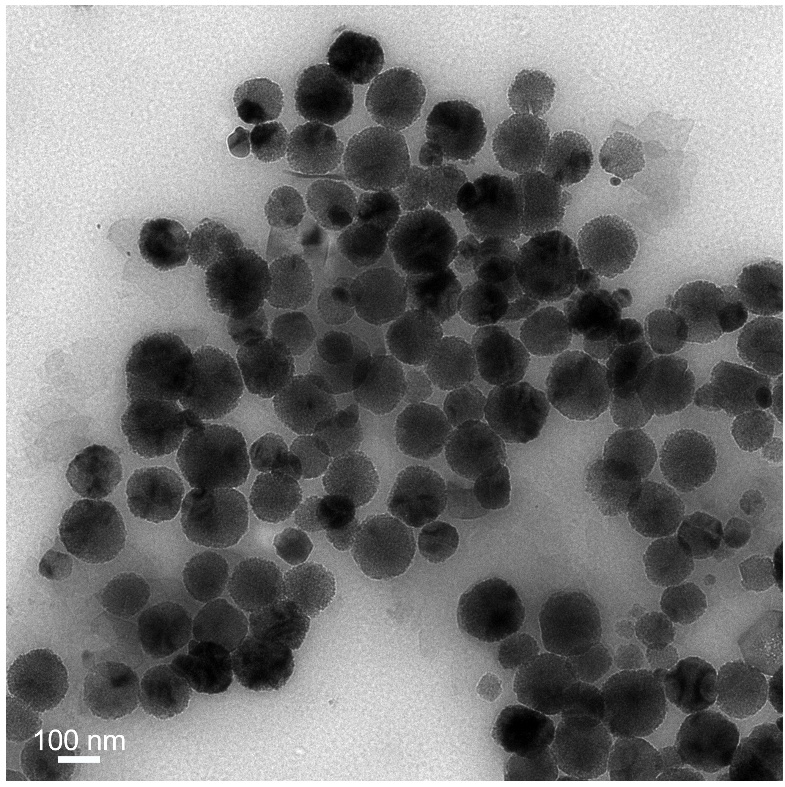


**Fig. S1** TEM image of Fe_3_O_4_ nanoparticles


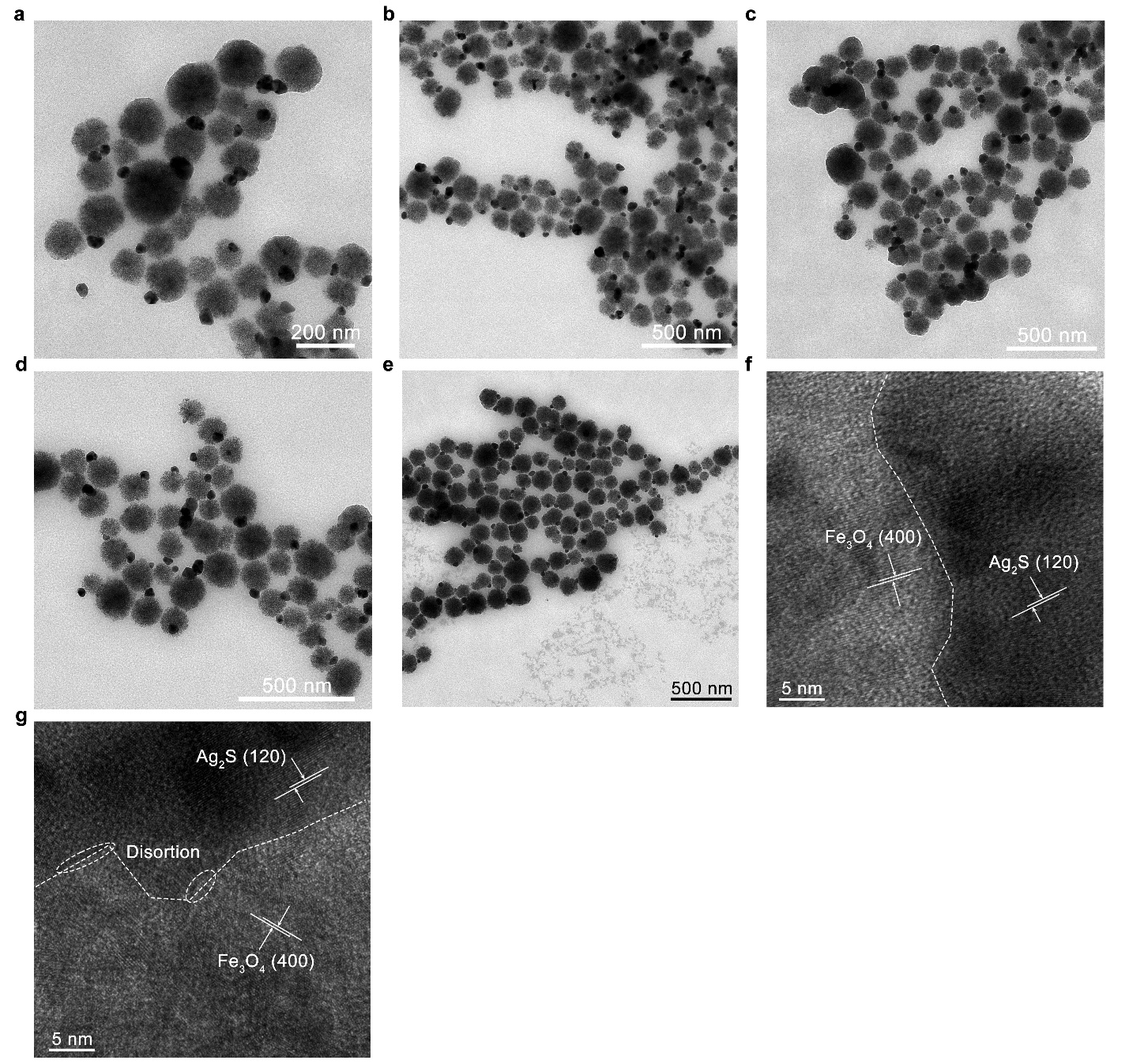


**Fig. S2** TEM images of **a-b** Fe_3_O_4_-Ag and **c-d** Fe_3_O_4_-Ag_2_S nanoparticles in various resolution. **e** TEM image of Fe_3_O_4_-Ag_2_S nanoparticles contained in medium with serum. HRTEM images of Fe_3_O_4_-Ag_2_S nanoparticles’ interfaces of Fe_3_O_4_ and Ag_2_S, **f** in PBS and **g** medium with serum cultured after 48 h


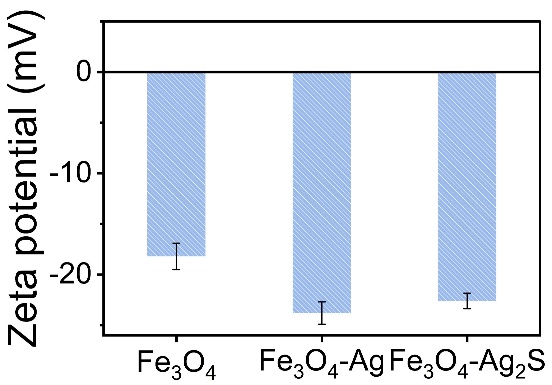


**Fig. S3** Zeta potential of Fe_3_O_4_, Fe_3_O_4_-Ag, and Fe_3_O_4_-Ag_2_S, data were presented as mean ± S.D. (*n* = 3)


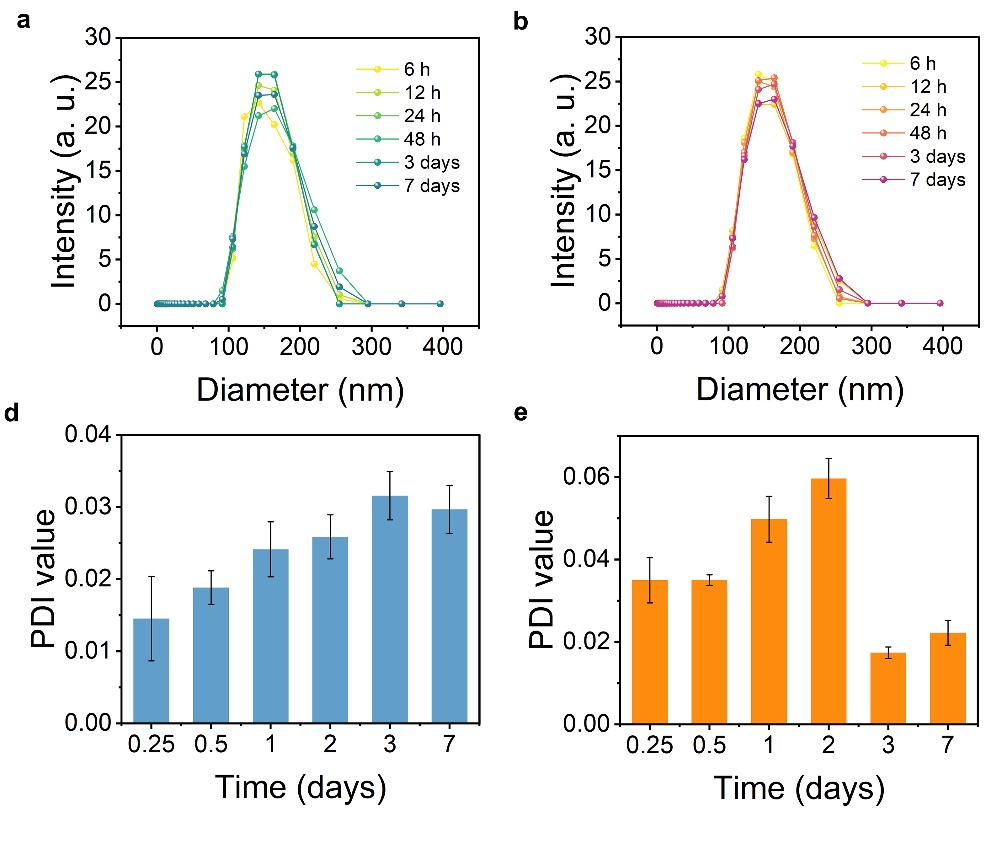


**Fig. S4** Dynamic light scattering patterns of Fe_3_O_4_-Ag_2_S over a 6, 12, 24, 48, 72, h and 7-days period in complete cell culture medium (containing 10% serum) in pH **a, d** 6 and **b, e** 7, data were presented as mean ± S.D. (*n* = 3)


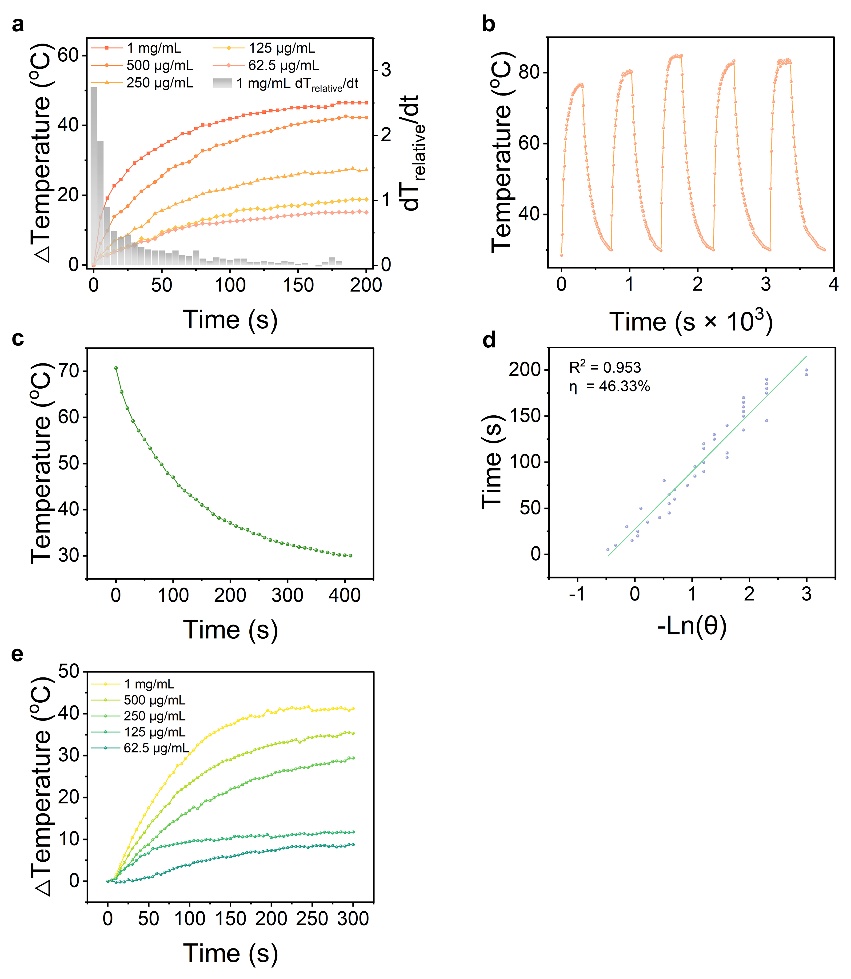


**Fig. S5 a** Time-dependent photothermal effect of Fe_3_O_4_-Ag_2_S. **b** Photothermal stability curve. **c** Cooling period, and **d** photothermal conversion efficiency. **e** Photothermal effect of Fe_3_O_4_


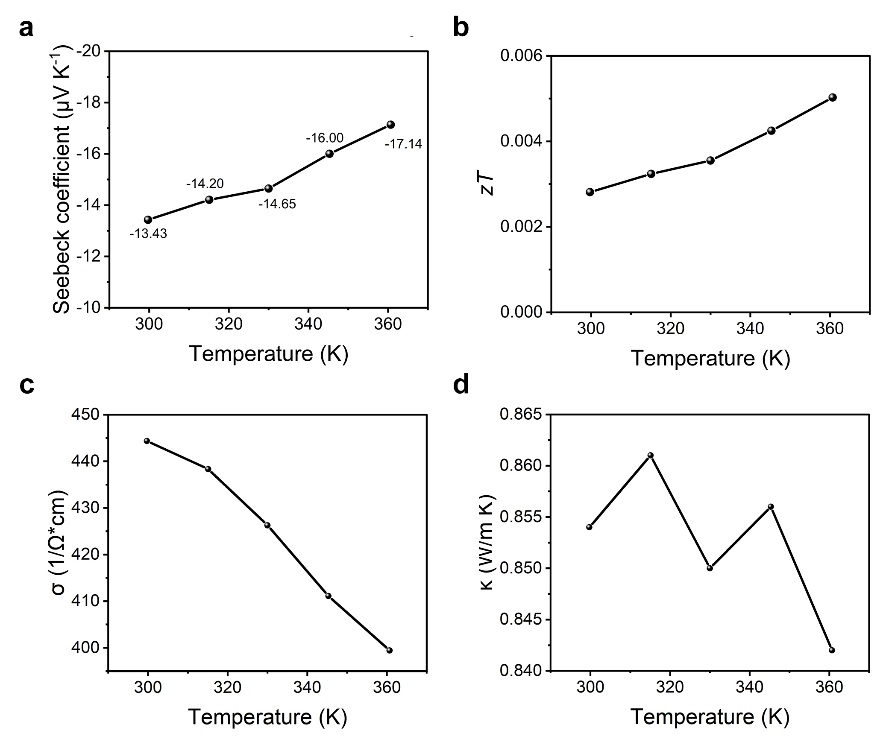


**Fig. S6** Temperature dependent **a** Seebeck coefficient, **b** calculated zT, **c** electrical conductivity, and **d** thermal conductivity of Fe_3_O_4_-Ag_2_S


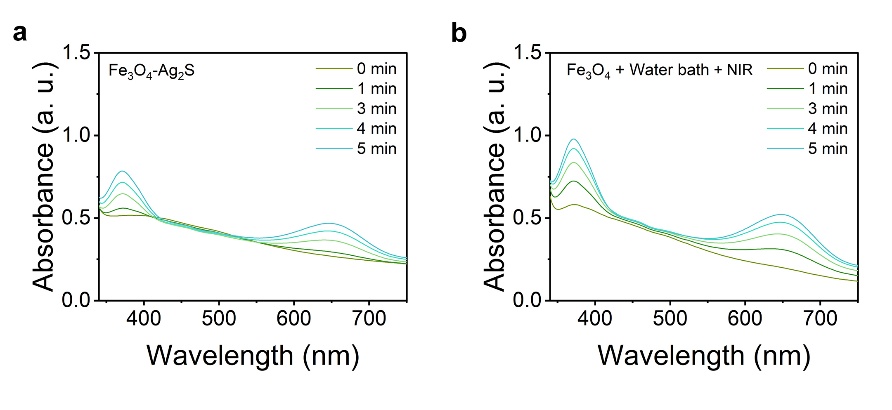


**Fig. S7** TMB indicated •OH generation of **a** Fe_3_O_4_-Ag_2_S and **b** Fe_3_O_4_ + Water bath + NIR


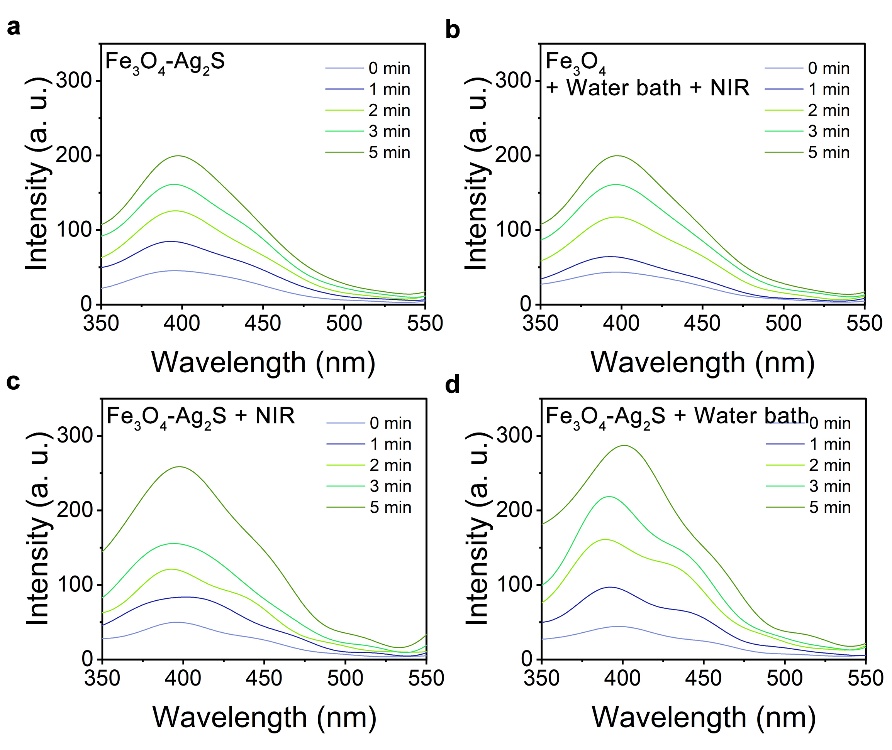


**Fig. S8** TA indicated •OH generation of **a** Fe_3_O_4_-Ag_2_S, **b** Fe_3_O_4_ + Water bath + NIR, **c** Fe_3_O_4_-Ag_2_S + NIR, and **d** Fe_3_O_4_-Ag_2_S + Water bath


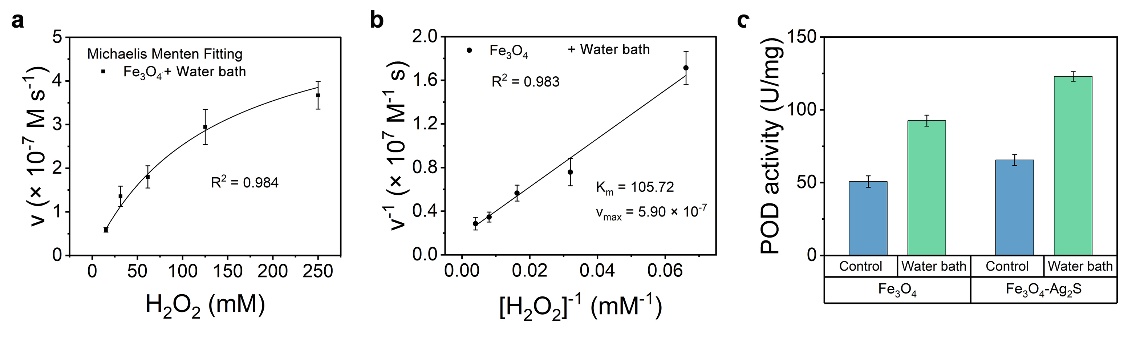


**Fig. S9 a** Michaelis-Menten kinetic analysis of Fe_3_O_4_ + Water bath and **b** Lineweaver-Burk plot, data were presented as mean ± S.D. (*n* = 3). **c** The enzymatic activities of Fe_3_O_4_ and Fe_3_O_4_-Ag_2_S determined by guaiacol method under room temperature and water bath conditions, data were presented as mean ± S.D. (*n* = 3)


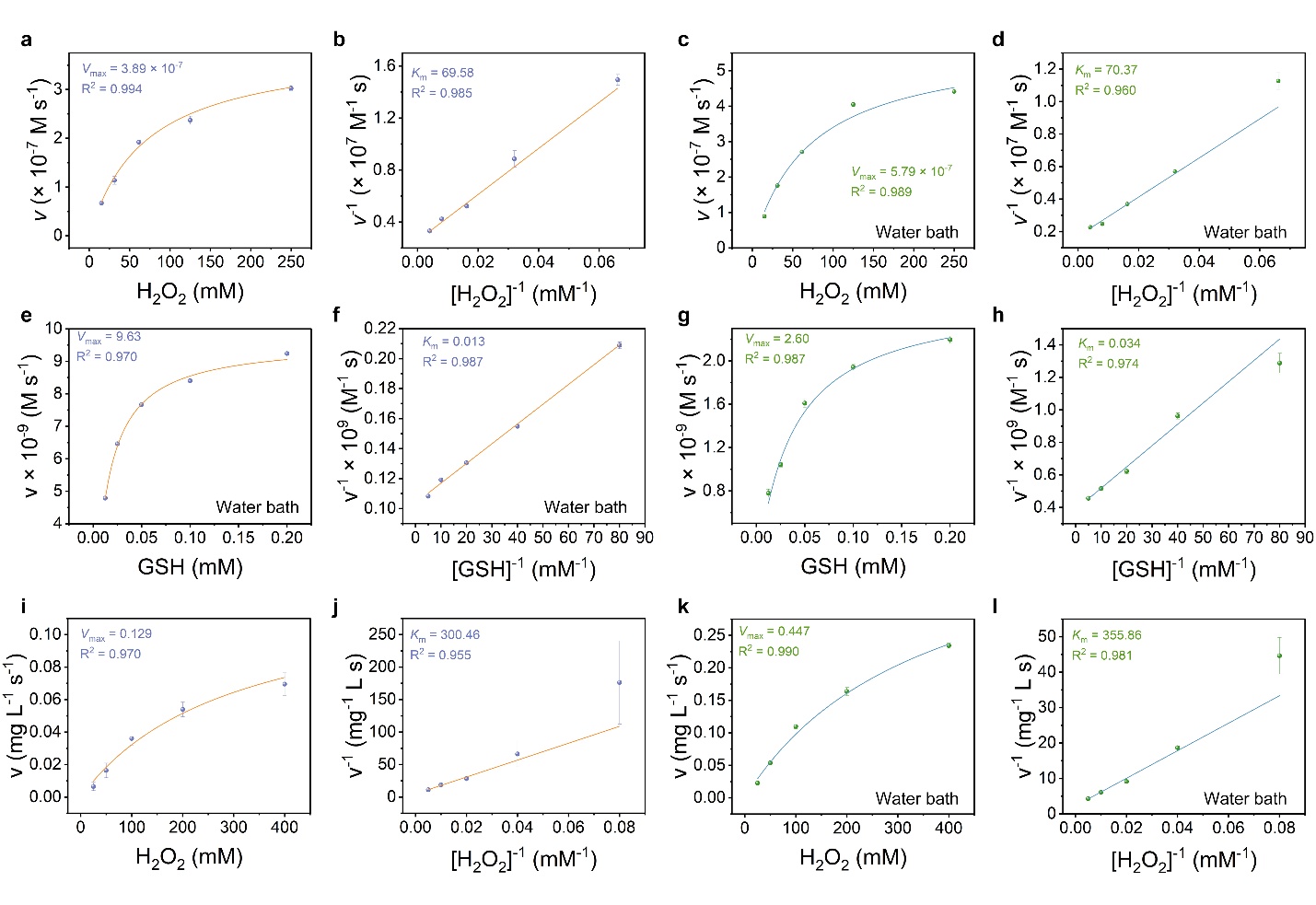


**Fig. S10 a-d** POD, **e-h** GSHox, and **i-l** CAT enzymatic activity of Fe_3_O_4_ + Ag_2_S physical mixture under room temperature or 60℃ water bath


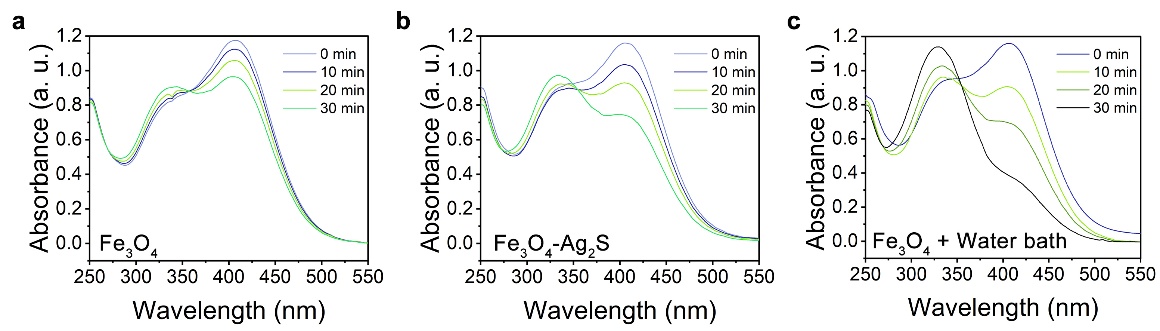


**Fig. S11** DTNB indicated GSH consumption of **a** Fe_3_O_4_, **b** Fe_3_O_4_-Ag_2_S, and **c** Fe_3_O_4_ + Water bath


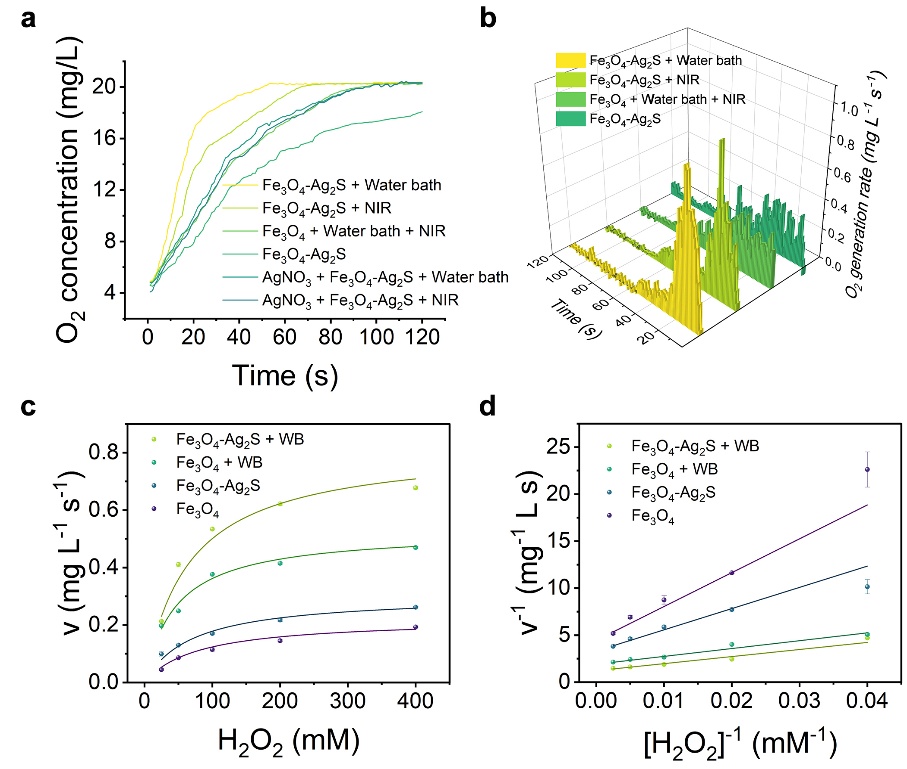


**Fig. S12 a** Time-dependent oxygen generation curves and **b** their differentiation under different conditions. **c** CAT enzyme analysis using Michaelis-Menten kinetic methods of Fe_3_O_4_ and Fe_3_O_4_-Ag_2_S with or without water bath and **d** Lineweaver-Burk plot, data were presented as mean ± S.D. (*n* = 3)


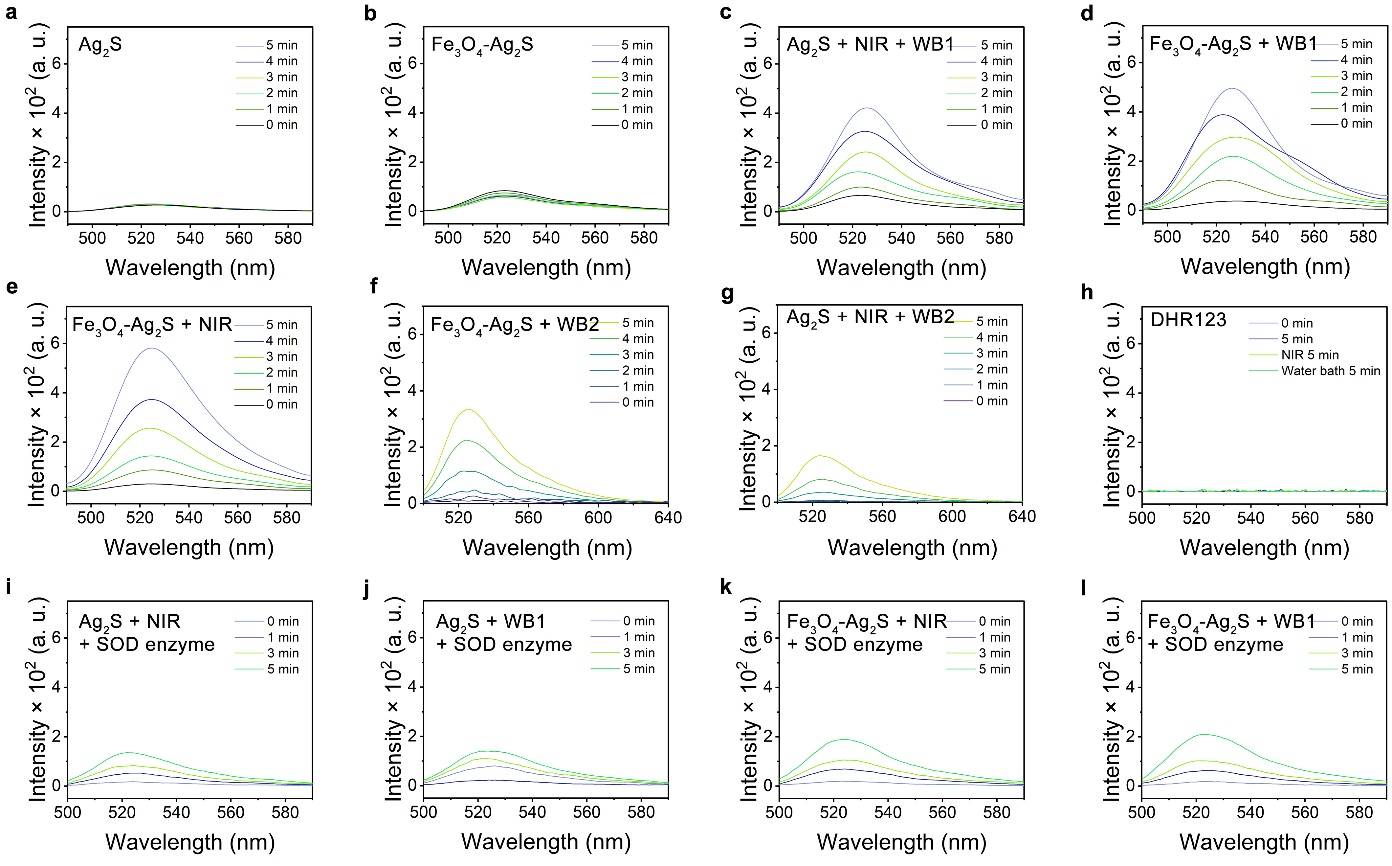


**Fig. S13** DHR123 indicated O_2_^•−^ generation at **a** Ag_2_S, **b** Fe_3_O_4_-Ag_2_S, **c** Ag_2_S + NIR + Water bath (80/30^o^C), **d** Fe_3_O_4_-Ag_2_S + Water bath (80/30^o^C), **e** Fe_3_O_4_-Ag_2_S + NIR, **f** Fe_3_O_4_-Ag_2_S + Water bath (60/30^o^C), **g** Ag_2_S + NIR + Water bath (60/30^o^C), **h** control group of pure DHR123, **i** Ag_2_S + NIR + SOD enzyme, **j** Ag_2_S + Water bath (80/30^o^C) + SOD enzyme, **k** Fe_3_O_4_-Ag_2_S + NIR + SOD enzyme, and **l** Fe_3_O_4_-Ag_2_S + Water bath (80/30^o^C) + SOD enzyme.


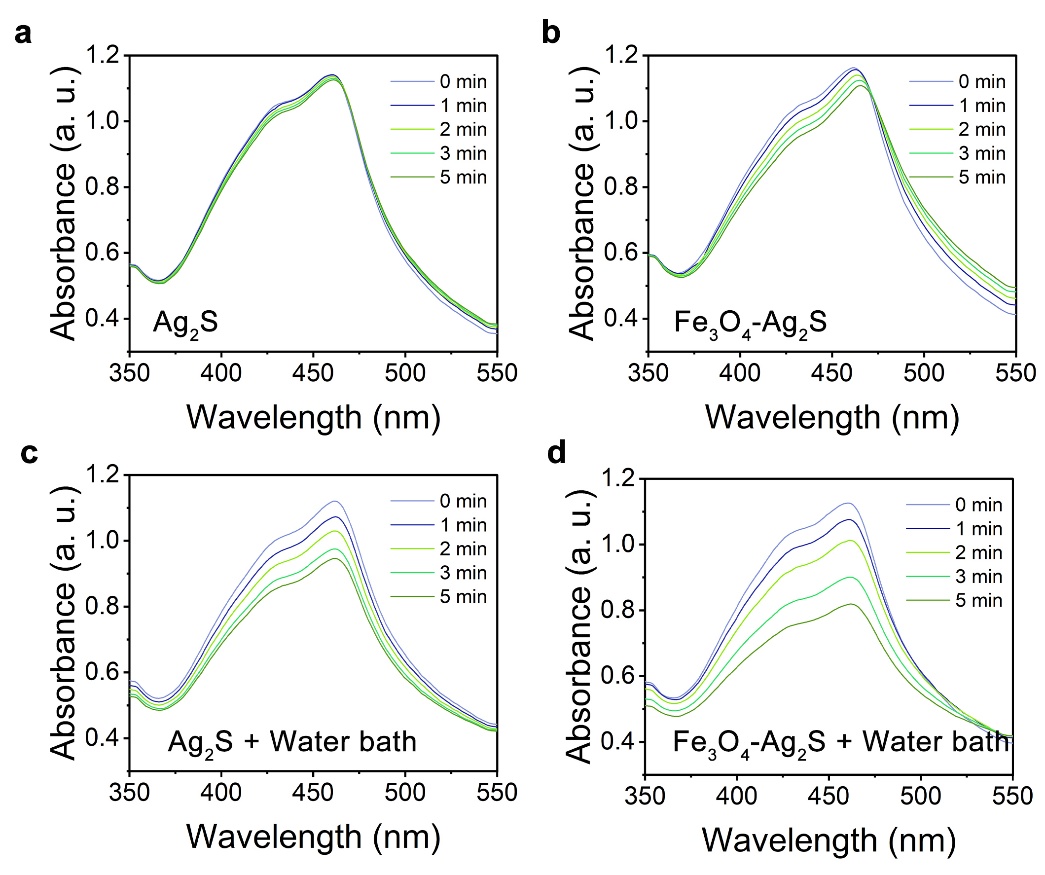


**Fig. S14** DPBF indicated ^1^O_2_ generation of **a, c** Ag_2_S and **b, d** Fe_3_O_4_-Ag_2_S with or without water bath


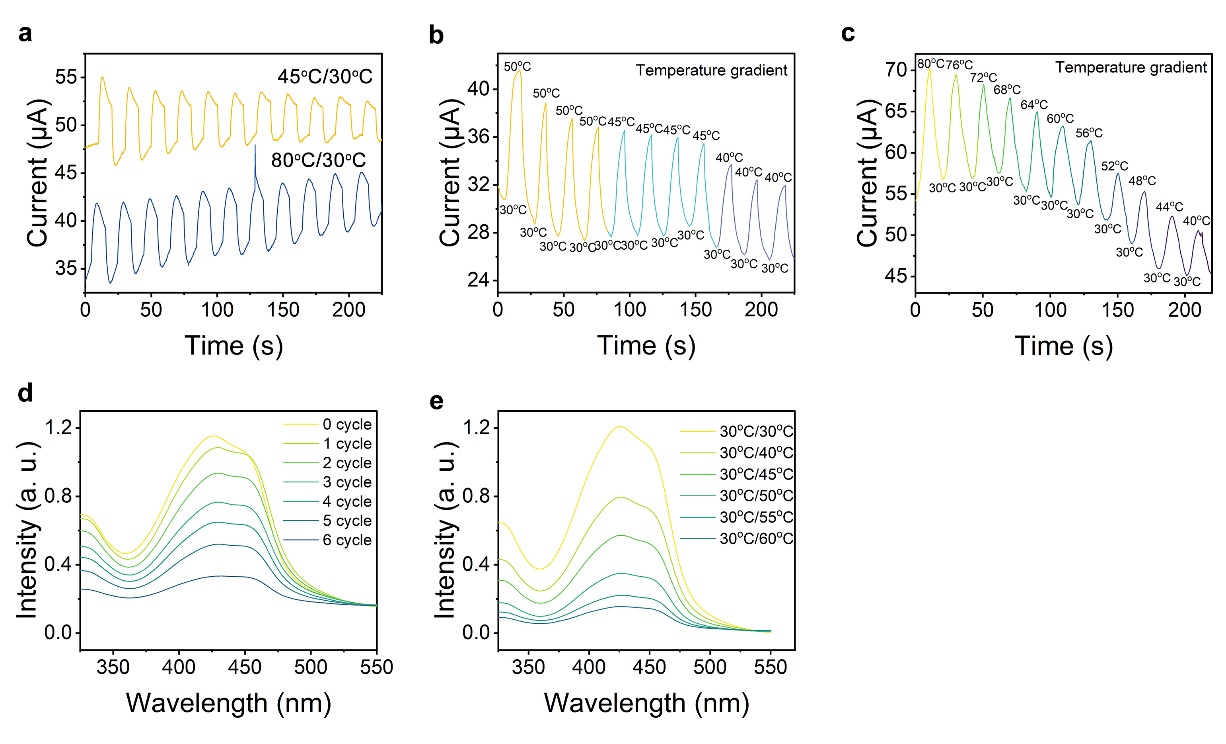


**Fig. S15 Fe_3_O_4_-Ag_2_S thermoelectric response.** **a** Current response under 80/30^o^C and 45/30^o^C water bath. **b** Current response under a temperature difference of 20, 15, and 10^o^C. **c** Current response under continuously changing temperature gradients. DPBF indicated ^1^O_2_ generation of Fe_3_O_4_-Ag_2_S under **d** a 30^o^C temperature gradient and **e** various temperature gradient


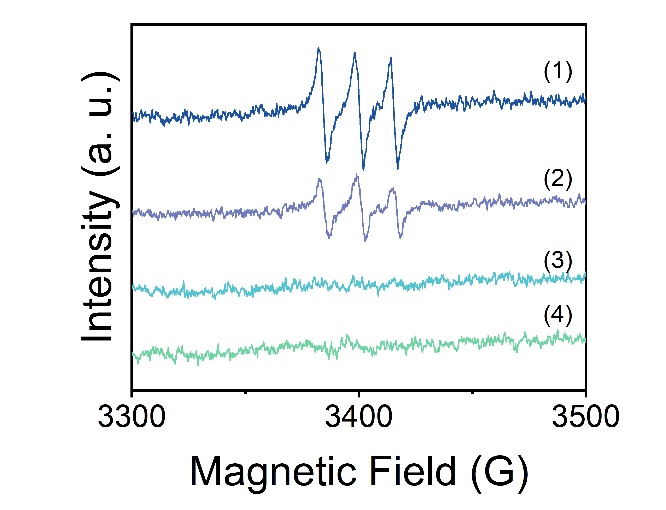


**Fig. S16** ESR spectra of ^1^O_2_ under different conditions. (1) Fe_3_O_4_-Ag_2_S + H_2_O_2_ + NIR in aqueous, (2) Fe_3_O_4_-Ag_2_S + H_2_O_2_ + AgNO_3_ + NIR in aqueous, (3) Fe_3_O_4_-Ag_2_S + AgNO_3_ + H_2_O_2_ + NIR in methanol and (4) Fe_3_O_4_-Ag_2_S + AgNO_3_ + NIR in methanol


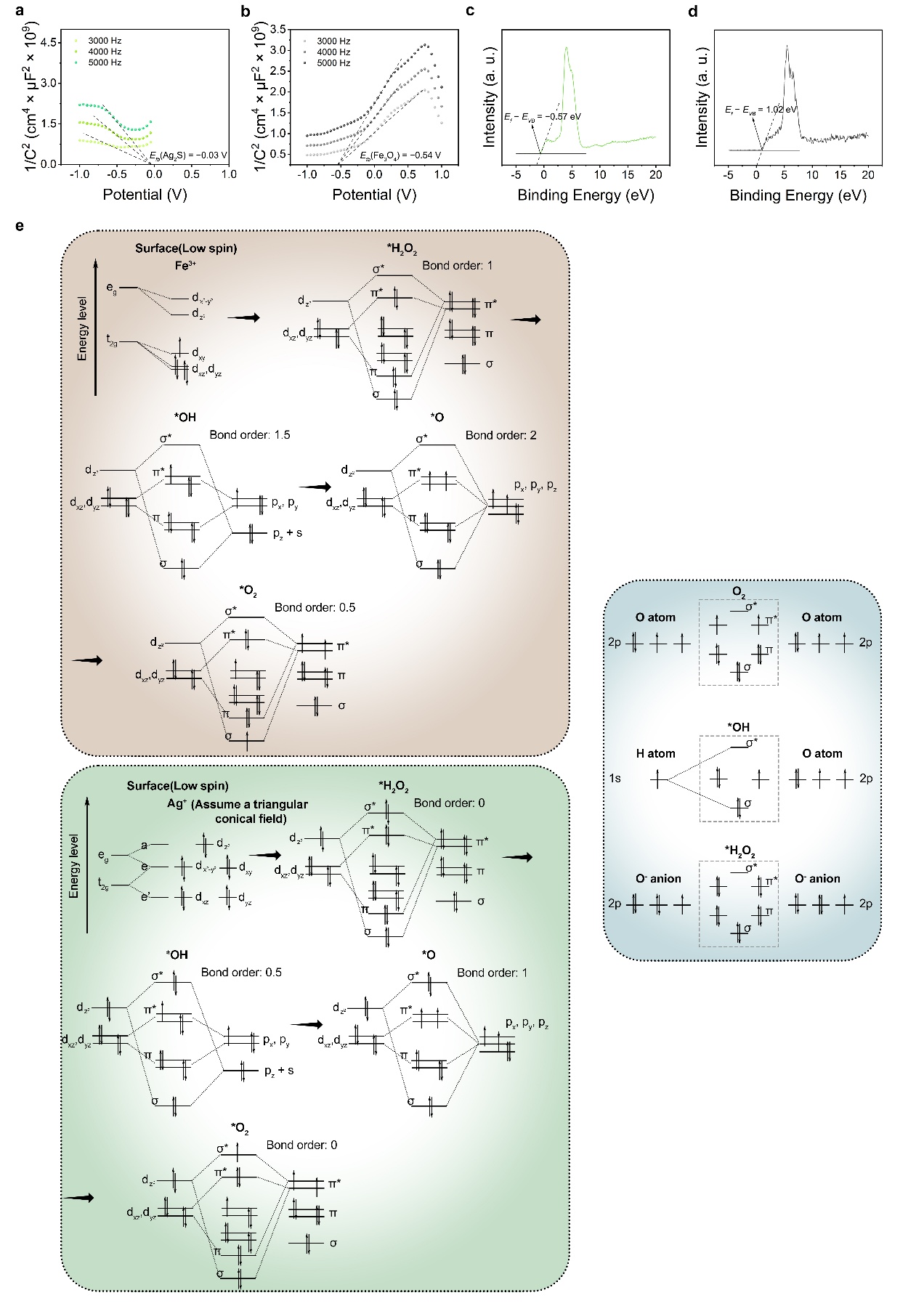


**Fig. S17** Mott-Schottky curves and XPS valence band spectra of **a, c** Ag_2_S and **b, d** Fe_3_O_4_. **e** Oxygen precipitation reaction history analyzed by molecular orbital theory of Fe^3+^ at the lowest spin state and Ag^+^


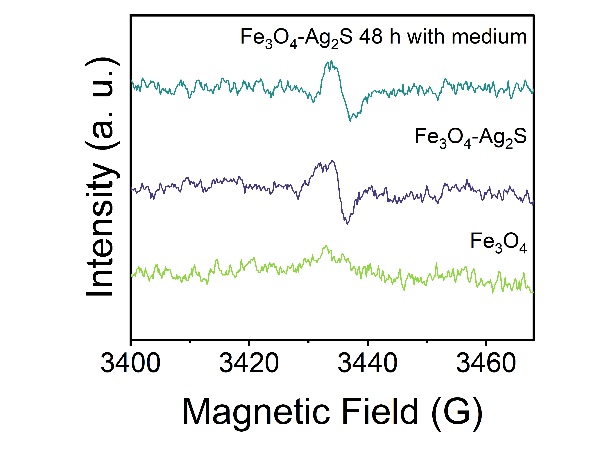


**Fig. S18** EPR spectrum of Fe_3_O_4_-Ag_2_S and Fe_3_O_4_. EPR spectrum of Fe_3_O_4_-Ag_2_S cultured with medium after 48 h


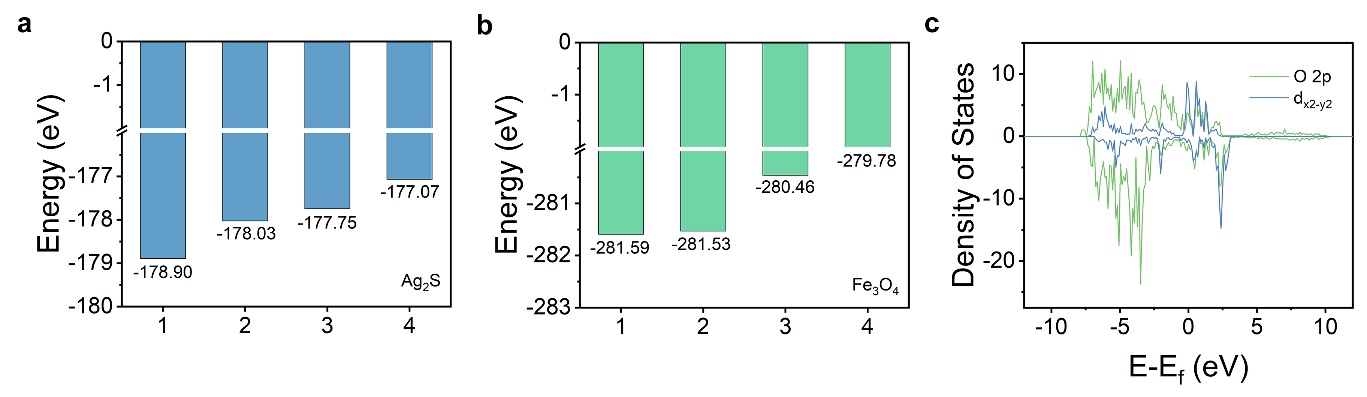


**Fig. S19** The energies of different surface termination modes of **a** Ag_2_S and **b** Fe_3_O_4_. **c** PDOS curves of O 2p and Fe d_x_^2^_-y_^2^ orbitals


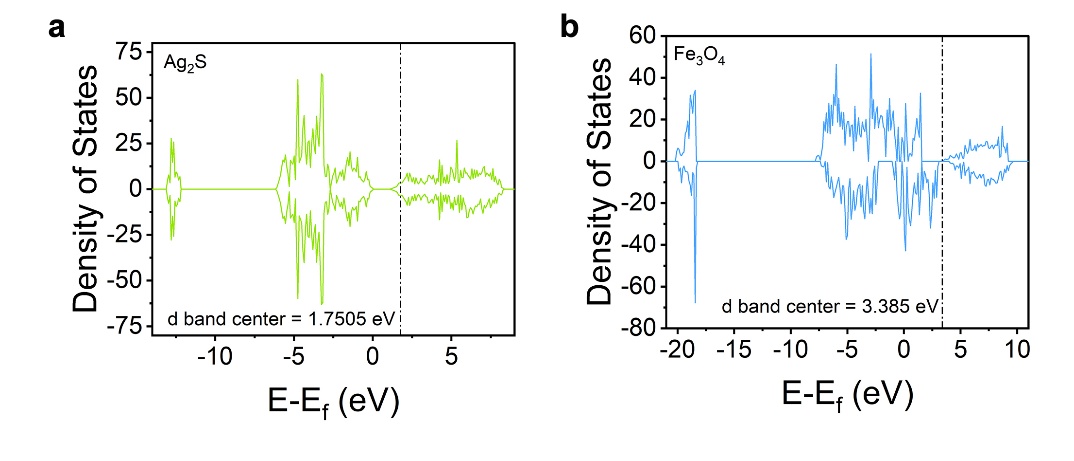


**Fig. S20** DOS curves and d band center of **a** Ag_2_S and **b** Fe_3_O_4_-Ag_2_S


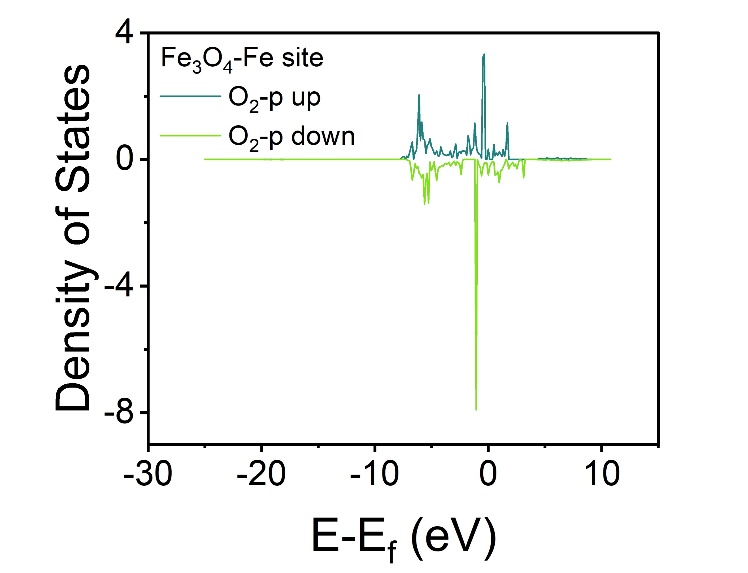


**Fig. S21** The PDOS of the p orbital of O_2_ adsorbed at the Fe site of Fe_3_O_4_


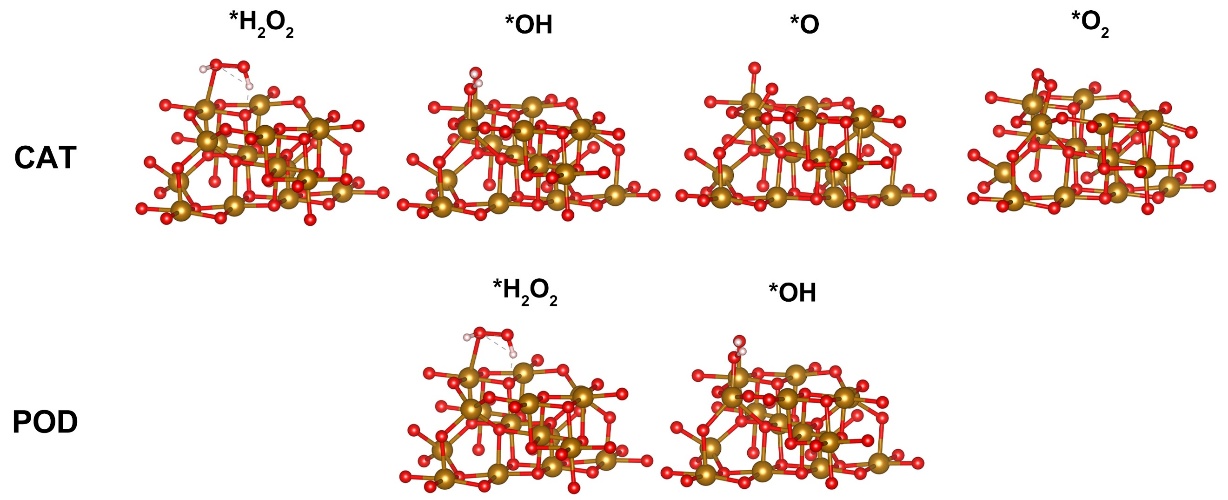


**Fig. S22** Surface configurations of different modeled initial, transition, and final states at Fe_3_O_4_ for the simulated CAT-like and POD-like catalytic processes


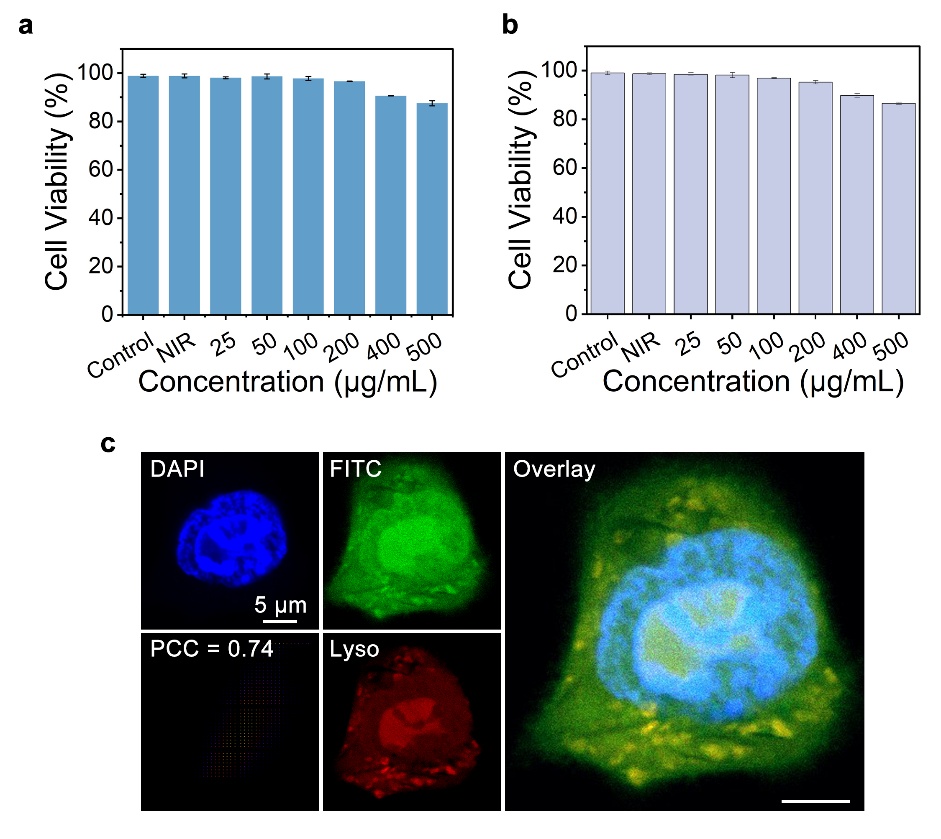


**Fig. S23** Biocompatibility of different Fe_3_O_4_-Ag_2_S concentrations with the **a** L929 fibroblast cell line and **b** mouse skeletal muscle myoblast cell line C2C12 in co-culture indication, data were presented as mean ± S.D. (*n* = 3). **c** Subcellular colocalization experiments of 4T1 cells and Fe_3_O_4_-Ag_2_S after co-culture for 12 hours


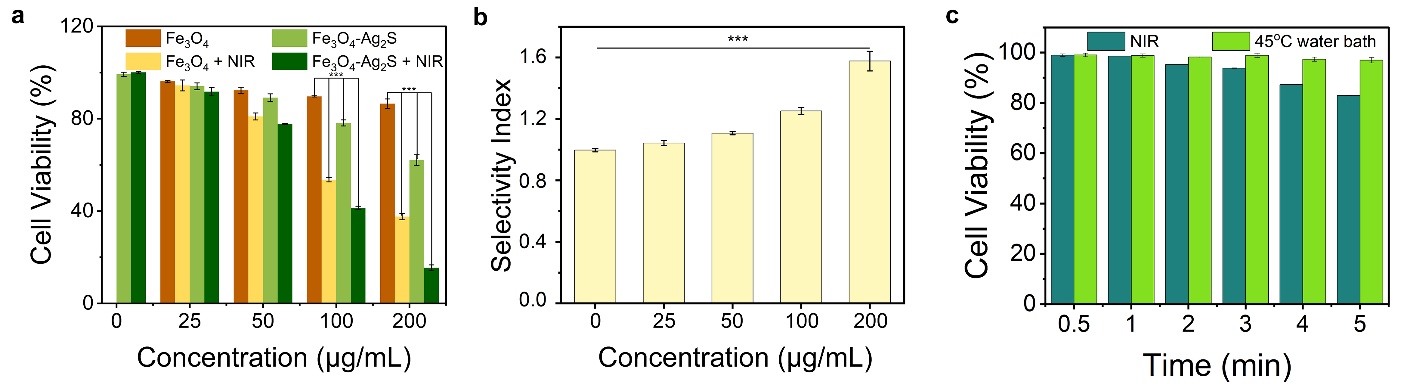


**Fig. S24 a** 4T1 cytotoxicity of different treatments as indicated by the MTT assay, data were presented as mean ± S.D. (*n* = 3), ****p* < 0.001. **b** Selectivity Index of Fe_3_O_4_-Ag_2_S in different concentrations. **c** 4T1 cell viability under NIR or 45^o^C water bath


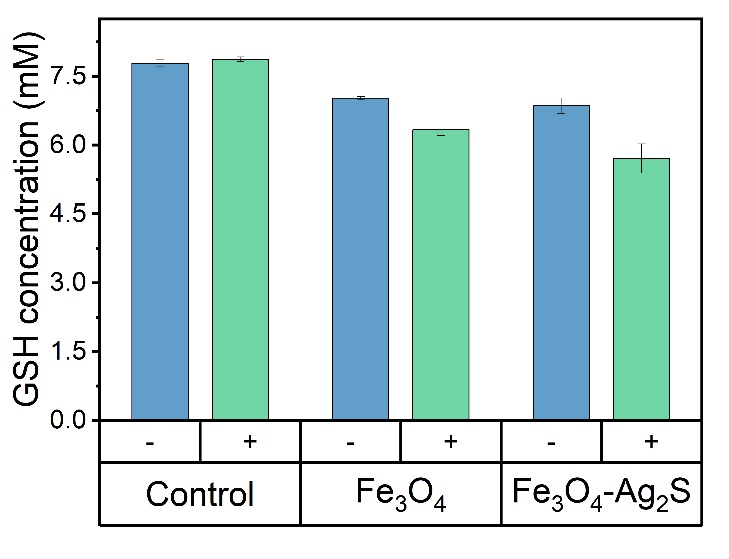


**Fig. S25** GSH concentration in 4T1 tumor cells under different treatment (+ and – represent to NIR on and off, respectively), data were presented as mean ± S.D. (*n* = 3)


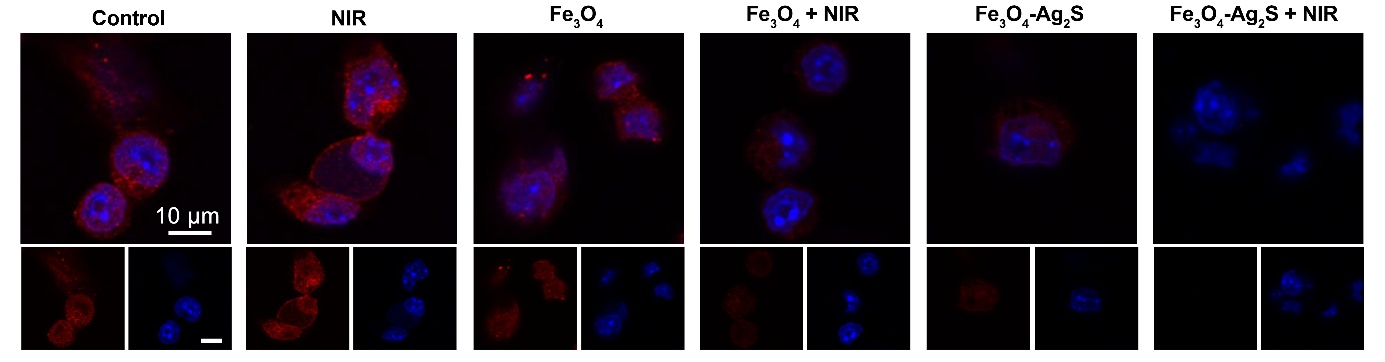


**Fig. S26** GPX4 immunofluorescent CLSM images in 4T1 tumor cells under different treatment


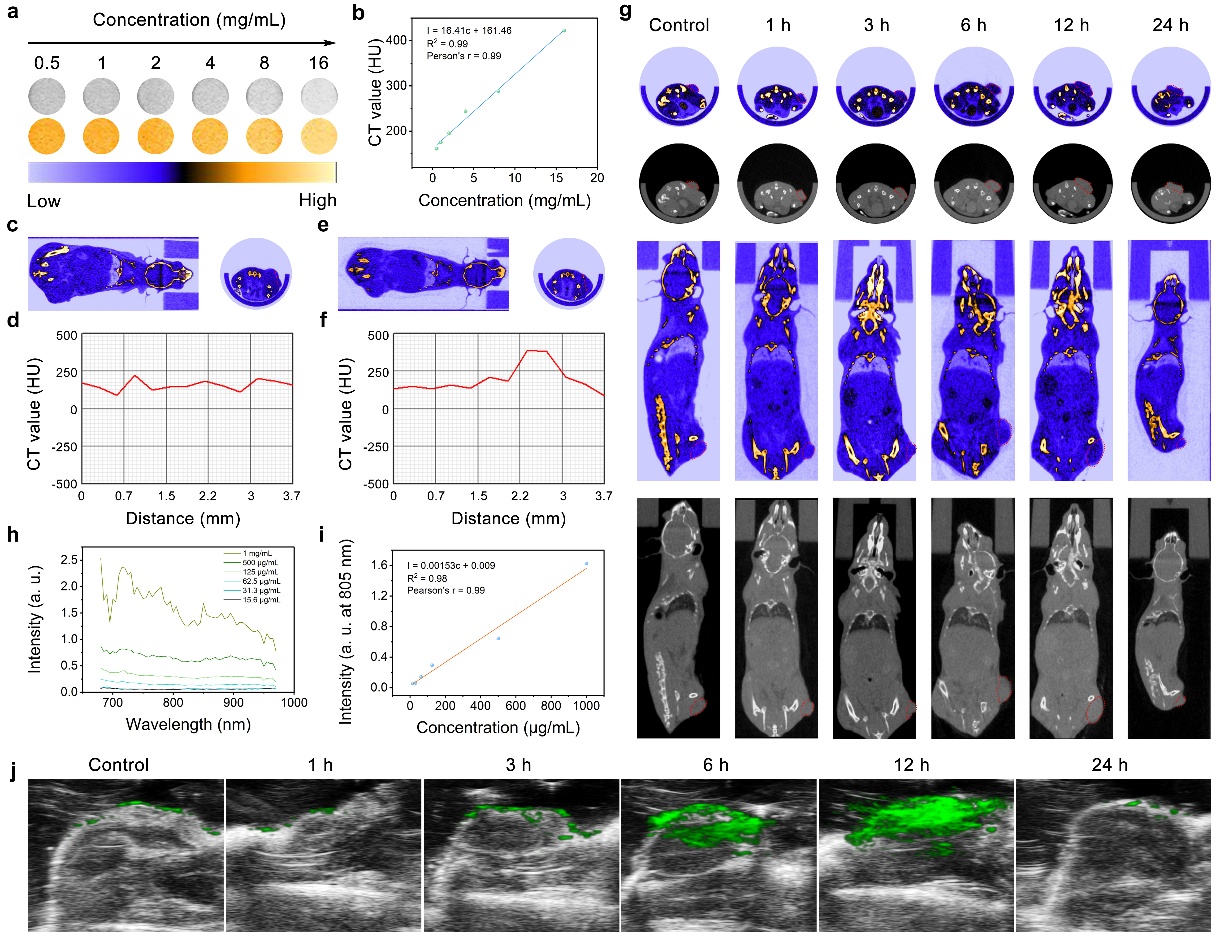


**Fig. S27** *In vitro* and *in vivo* CT and PA Imaging. **a** *In vitro* CT images and pseudo-color images of Fe_3_O_4_-Ag_2_S solutions with different concentrations in PBS. **b** CT value plots of Fe_3_O_4_-Ag_2_S with the concentrations. *In vivo* CT images of tumor-bearing mice before **c** and after **e** *i.t.* injection of Fe_3_O_4_-Ag_2_S (20 mg mL^−1^), and **d, f** the corresponding cross-sectional compositional line profiles of CT value along the line. **g** *In vivo* CT images after *i.v.* injection of Fe_3_O_4_-Ag_2_S solution at different times. **h** PA signal-excitation wavelength curves for various concentrations Fe_3_O_4_-Ag_2_S. **i** PA signal intensity plots of Fe_3_O_4_-Ag_2_S with the concentrations. **j** *In vivo* PA images after *i.v.* injection of Fe_3_O_4_-Ag_2_S solution at different times


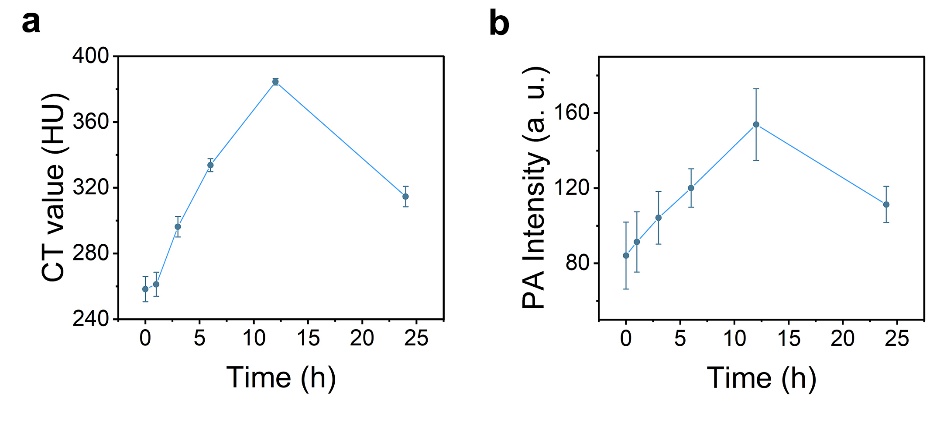


**Fig. S28** In vivo **a** CT and **b** PA signal analysis, data were presented as mean ± S.D. (*n* = 3)


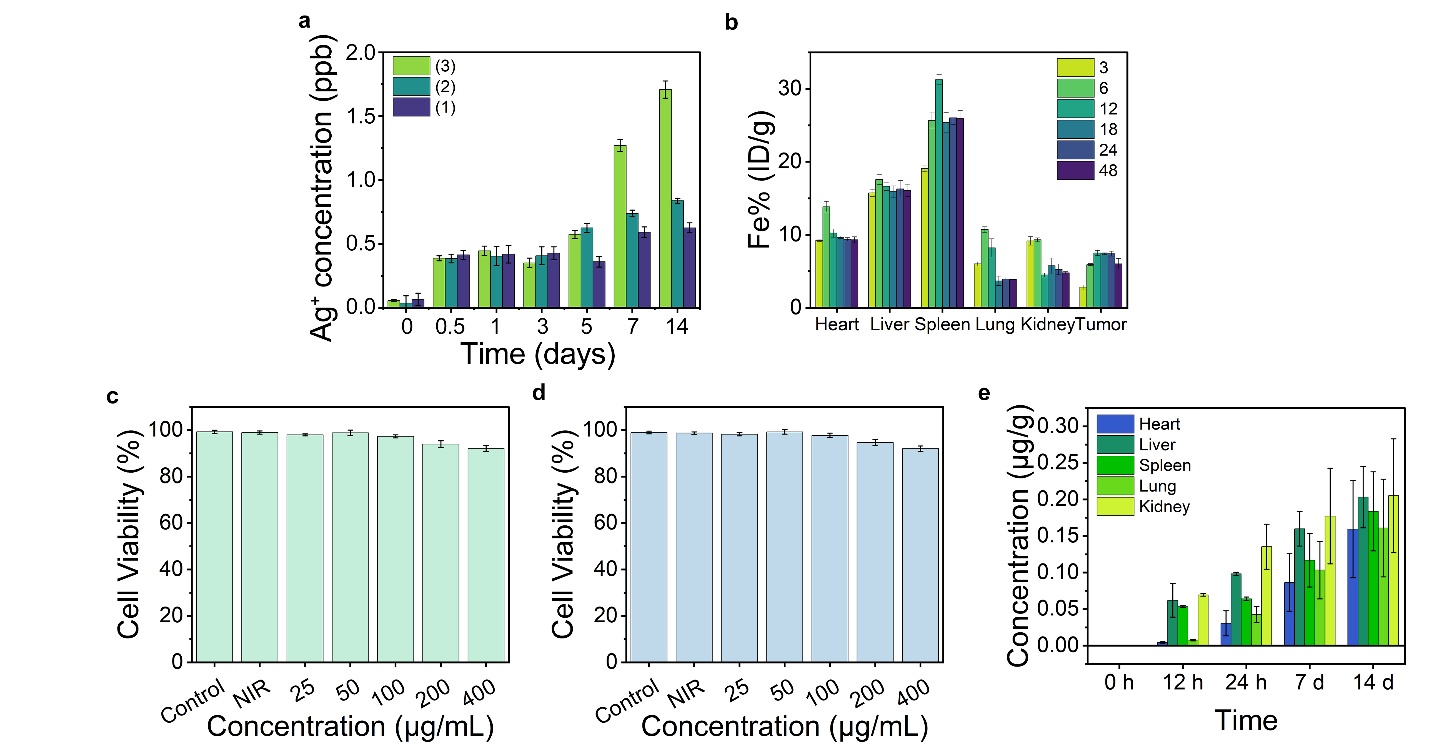


**Fig. S29 a** Ag^+^ release kinetic analysis using ICP-MS under different solvent: (1) Saline, pH 6.5, with 100 μM H_2_O_2_ and 100 μM GSH; (2) PBS, pH 6.5, with 100 μM H_2_O_2_ and 100 μM GSH; (3) Medium, pH 6.5, with 100 μM H_2_O_2_ and 100 μM GSH, during 0.5, 1, 3, 5, 7, and 14 days. **b** ICP-OES indicated drug distributions during during 3, 6, 12, 18, 24, and 48 h using Fe element distribution. MTT assay of **c** HL-7702 hepatocytes and **d** HK-2 renal proximal tubular cells. **e** The accumulation of Ag^+^ in various organs at different times after *i.v.* injection of Fe_3_O_4_-Ag_2_S, data were presented as mean ± S.D. (*n* = 3)


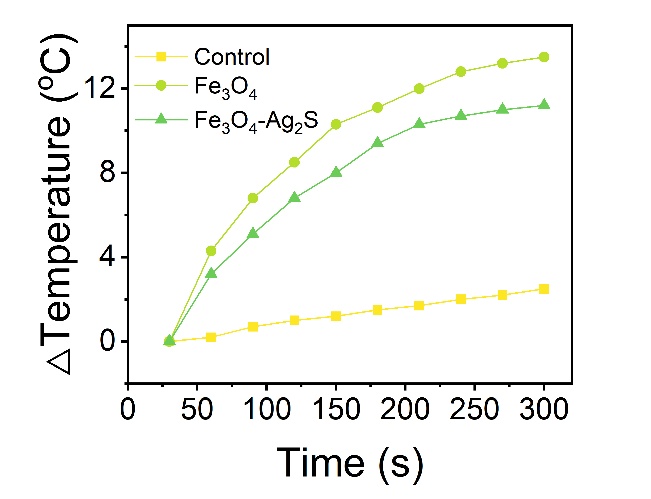


**Fig. S30** *In vivo* photothermal property of PBS (Control), Fe_3_O_4_, and Fe_3_O_4_-Ag_2_S


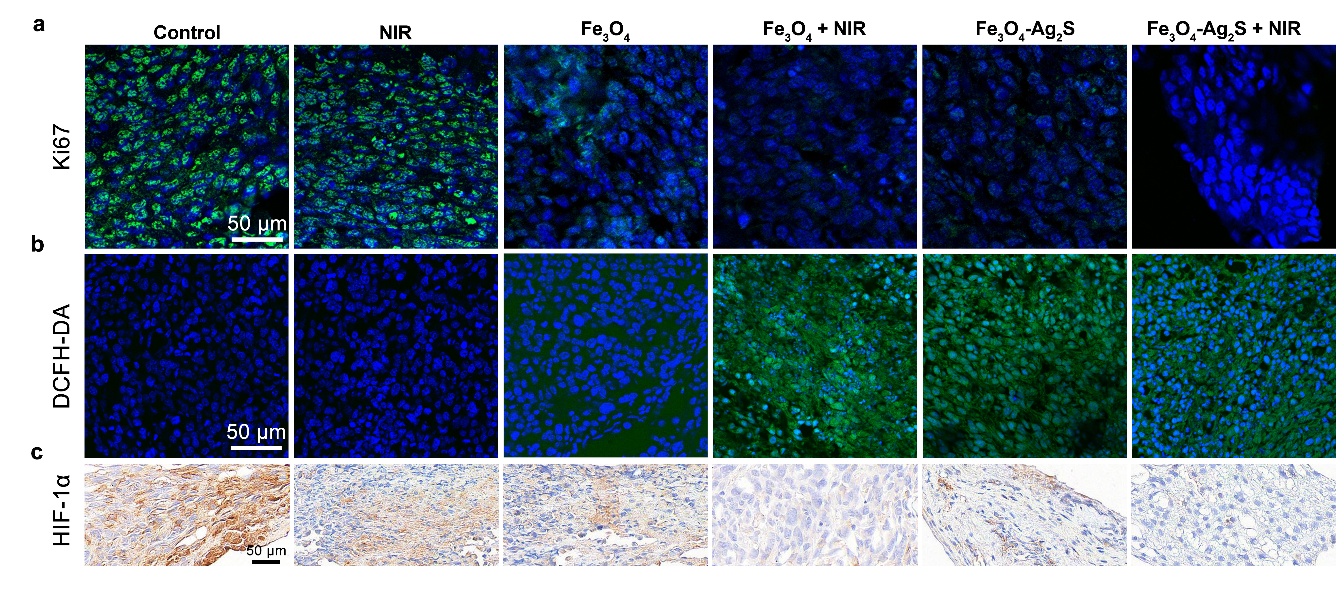


**Fig. S31 a** Ki67 staining. **b** Intratumoral ROS concentration indicated by DCFH-DA staining. **c** HIF-1α immunohistochemical staining


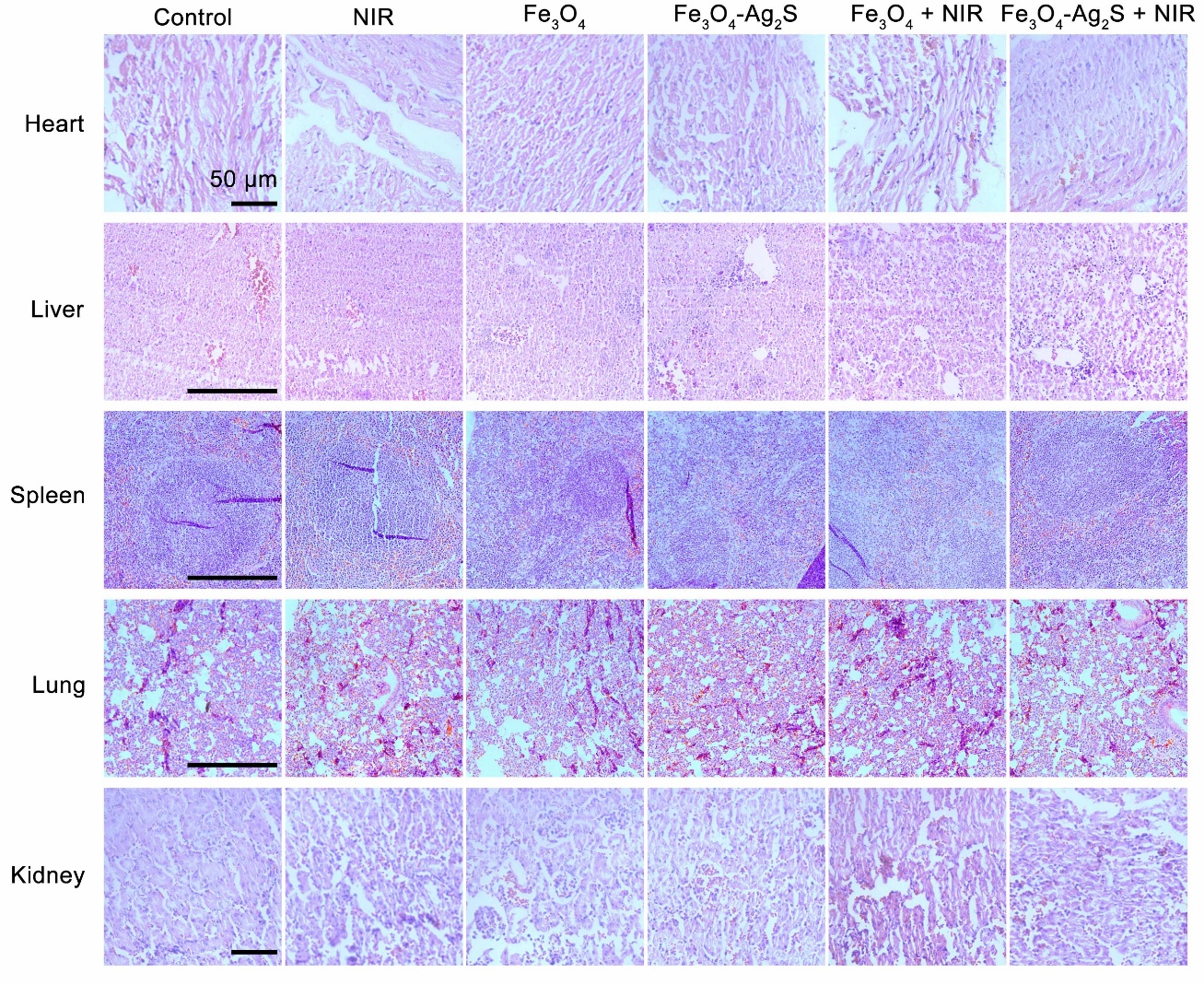


**Fig. S32** H&E staining of heart, liver, spleen, lung, and kidney tissue sections


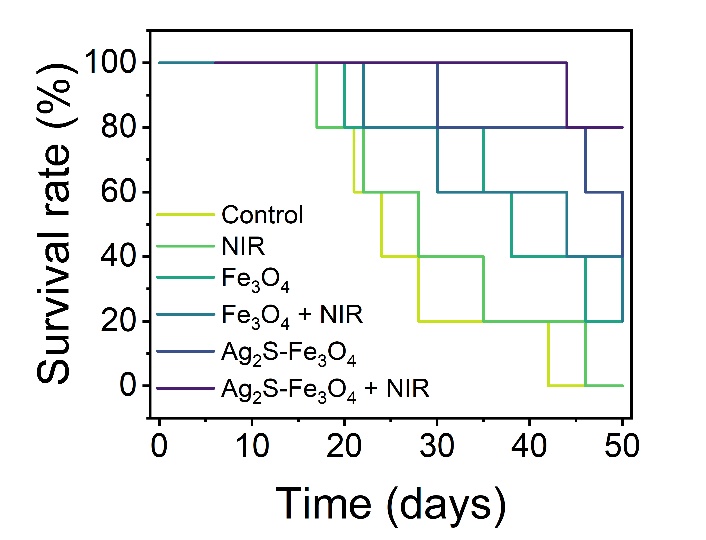


**Fig. S33** The Kaplan-Meier survival curves of each treatment group
